# Engineering *Escherichia coli* to grow constitutively on D-xylose using the carbon-efficient Weimberg pathway

**DOI:** 10.1099/mic.0.000611

**Published:** 2018-02-05

**Authors:** Luca Rossoni, Reuben Carr, Scott Baxter, Roxann Cortis, Thomas Thorpe, Graham Eastham, Gill Stephens

**Affiliations:** ^1^​ University of Nottingham, Bioprocess, Environmental and Chemical Technologies Research Group, Nottingham, UK; ^2^​ Ingenza Ltd, Edinburgh, UK; ^3^​ Lucite International, Wilton, UK

**Keywords:** pentose utilization, D-xylose, Weimberg pathway, constitutive growth

## Abstract

Bio-production of fuels and chemicals from lignocellulosic C5 sugars usually requires the use of the pentose phosphate pathway (PPP) to produce pyruvate. Unfortunately, the oxidation of pyruvate to acetyl-coenzyme A results in the loss of 33 % of the carbon as CO_2_, to the detriment of sustainability and process economics. To improve atom efficiency, we engineered *Escherichia coli* to utilize d-xylose constitutively using the Weimberg pathway, to allow direct production of 2-oxoglutarate without CO_2_ loss. After confirming enzyme expression *in vitro*, the pathway expression was optimized *in vivo* using a combinatorial approach, by screening a range of constitutive promoters whilst systematically varying the gene order. A PPP-deficient (*ΔxylAB*), 2-oxoglutarate auxotroph (*Δicd*) was used as the host strain, so that growth on d*-*xylose depended on the expression of the Weimberg pathway, and variants expressing *Caulobacter crescentus xylXAB* could be selected on minimal agar plates. The strains were isolated and high-throughput measurement of the growth rates on d-xylose was used to identify the fastest growing variant. This strain contained the pL promoter, with *C. crescentus xylA* at the first position in the synthetic operon, and grew at 42 % of the rate on d-xylose compared to wild-type *E. coli* using the PPP. Remarkably, the biomass yield was improved by 53.5 % compared with the wild-type upon restoration of *icd* activity. Therefore, the strain grows efficiently and constitutively on d-xylose, and offers great potential for use as a new host strain to engineer carbon-efficient production of fuels and chemicals *via* the Weimberg pathway.

## Introduction

Second-generation manufacturing of bio-based chemicals depends on the utilization of lignocellulosic wastes from food production and agriculture, to avoid any competition with human food supplies, a crucial requirement in a world where there is already insufficient food to feed a growing human population [1]. C5 sugars (e.g. d-xylose) account for a large proportion of all monosaccharides entrapped within lignocellulosic wastes [2, 3]. Therefore, efficient utilization of C5 sugars is a critical target for sustainable bio-production of chemicals and fuels [4–7].

**Fig. 1.**
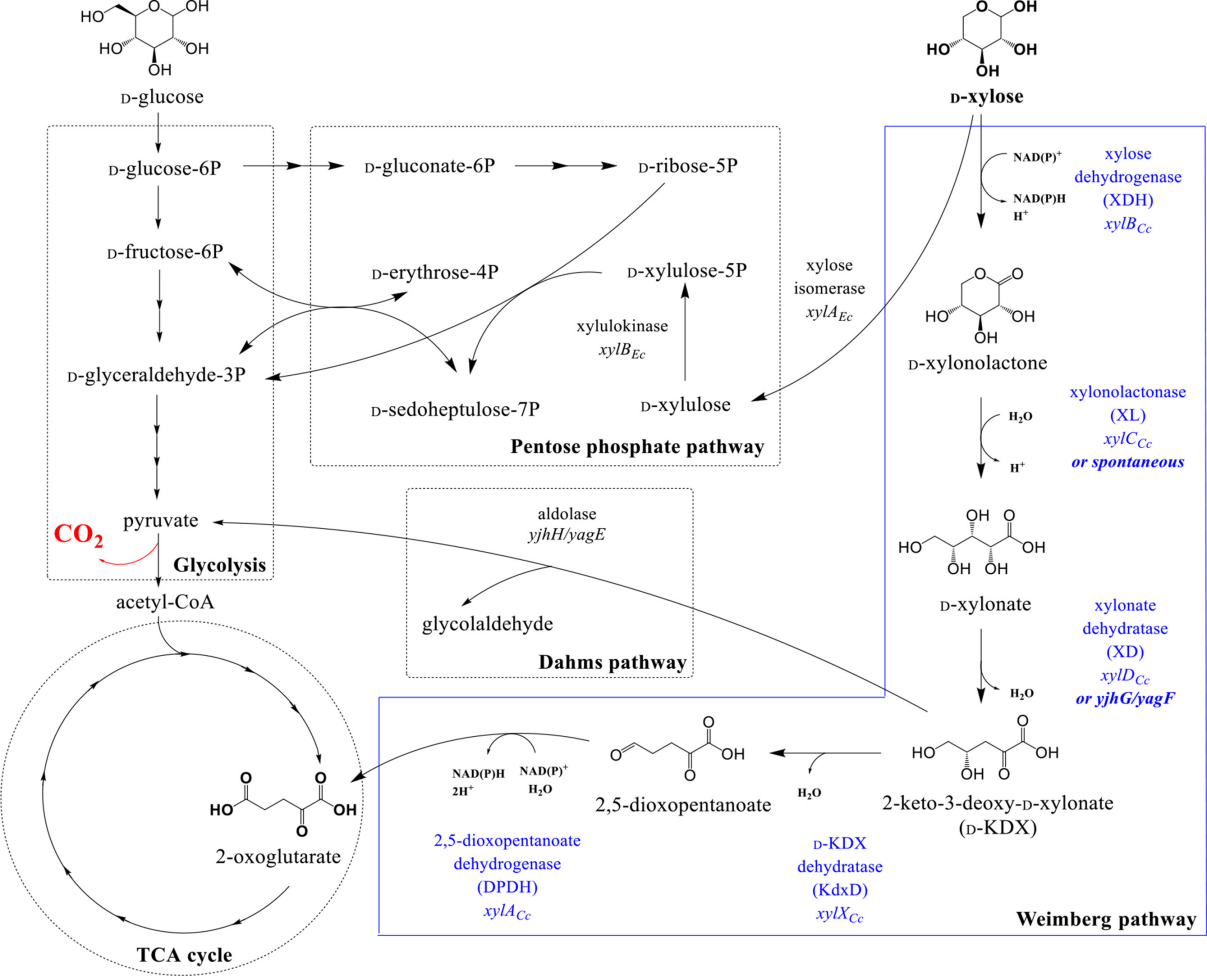
d-xylose assimilation pathways. The figure shows d-xylose assimilation *via* the pentose phosphate pathway, the Weimberg pathway and the Dahms pathway. The reactions of the pentose phosphate pathway and of the Dahms pathway are native in *E. coli*. The five reactions of the Weimberg pathway (blue box) require enzymes from *Caulobacter crescentus*, as indicated. The hydrolysis of xylonolactone can also occur spontaneously [30] and, in *E. coli*, the dehydration of xylonate is also catalyzed by the native *yjhG* and *yagF* gene activity [26–29].

In the conventional microbial strains used as cell factories (e.g. yeast and *Escherichia coli*), d-xylose is fermented *via* the pentose phosphate pathway (PPP). However, the engineered metabolic pathways used for chemical production often require the production of tricarboxylic acid (TCA) cycle intermediates, which are then elaborated to the final chemical products. The PPP results in the formation of pyruvate, which is then oxidized to acetyl-coenzyme A (acetyl-CoA), the substrate for the TCA cycle. Unfortunately, the production of acetyl-CoA also produces CO_2_, thus wasting 33 % of the substrate carbon (Fig. 1). Since atom efficiency is a key driver for the sustainability and economic viability of the chemical industry [8], we engineered an *E. coli* strain that can grow on lignocellulosic d-xylose constitutively *via* the Weimberg pathway.

The Weimberg pathway [9] allows the direct oxidation of d-xylose to 2-oxoglutarate, allowing direct conversion of the C5 skeleton to a C5 TCA cycle intermediate, without CO_2_ evolution (Fig. 1). The pathway is initiated by the conversion of d-xylose to d-xylonolactone, catalyzed by xylose dehydrogenase (XDH), followed by xylonolactonase (XL)-catalyzed hydrolysis and ring opening to form d-xylonate, and dehydration to 2-keto-3-deoxy-d-xylonate (d-KDX), catalyzed by xylonate dehydratase (XD). d-KDX dehydratase (KdxD) then catalyzes the formation of 2,5-dioxopentanoate, which is oxidized to 2-oxoglutarate by 2,5-dioxopentanoate dehydrogenase (DPDH).

The Weimberg pathway has already attracted significant attention as a means to develop carbon-efficient engineered metabolic pathways for bio-based production of chemicals. For example, upstream enzymes from the Weimberg pathway have been coupled with a decarboxylase from *Pseudomonas putida* or *Lactobacillus lactis* and native *E. coli* alcohol dehydrogenases or aldehyde dehydrogenases to produce d-1,2,4-butanetriol, d-1,4-butanediol and 3,4-dihydroxybutyric acid from d-xylose [10–16]. The complete pathway has also been used to produce glutaric acid and mesaconic acid using *E. coli* as the host strains, or to improve *Corynebacterium glutamicum* d-xylose utilization [17–21]. However, these systems relied on inducible expression, using an expensive inducer (IPTG), which increases the process costs. Furthermore, the engineered *E. coli* strains were dependent on glucose for growth in minimal media [17, 19], so that the benefit of using d-xylose to manufacture the chemicals is offset by the use of food-grade glucose to produce the *E. coli* biocatalyst. The engineered *C. glutamicum* strains could grow on d-xylose alone, but the growth rates were only a fraction of those achieved by the progenitor strains using the PPP [18, 20]. Therefore, we developed an *E. coli* strain that is able to grow efficiently and constitutively using the Weimberg pathway to oxidize d-xylose as the sole carbon and energy source. This new strain offers great potential as a novel host for future metabolic engineering to manufacture bio-based chemicals from lignocellulosic sugars.

## Methods

### Materials and growth media

The enzymes for gene cloning, plasmid purification kits and SDS-PAGE materials were purchased from Fermentas, Qiagen and Bio-Rad, respectively. 2-Keto-3-deoxy-d-xylonate (d-KDX) was synthesized using an established procedure [22], except for the preparation of the phosphonate silyl ester (Fig. S1, available in the online version of this article). All the other chemicals were purchased from Sigma-Aldrich.

The *E. coli* strains were cultured at 37 °C with shaking at 200 r.p.m. (unless specified otherwise) in either LB or ML medium. The ML medium contained (per L) (NH_4_)SO_4_ (2 g), K_2_HPO_4_ (14.6 g), NaH_2_PO_4_.2H_2_O (3.6 g) and NH_4_Cl (0.24 g) added from a 5× concentrated stock solution; MgSO_4_ (0.24 g) added from a 100×stock solution; and CaCl_2_.2H_2_O (1 mg), FeCl_3_ (20.06 mg), ZnSO_4_.7H_2_O (0.36 mg), CuSO_4_.5H_2_O (0.32 mg), MnSO_4_.H_2_O (0.30 mg), CoCl.6H_2_O (0.36 mg) and Na_2_EDTA.2H_2_O (44.6 mg) added from a 500× stock solution. d-Xylose or d-glucose (10 g l^−1^) were also added from stock solutions to produce ML-X and ML-G, respectively, and dH_2_O was used to adjust the volume to 1 l. Agar (15 g l^−1^) was added as required. All of the solutions were autoclaved before utilization. When required, kanamycin, carbenicillin and chloramphenicol were added from filter-sterilized stock solutions to final concentrations of 50, 50 and 34 mg l^−1^ to produce ML-XKm, ML-XCb or ML-XCm, or LB-Km, LB-Cb or LB-Cm, respectively. The stock cultures were prepared by mixing cultures growing exponentially in LB (0.85 ml) with sterile 80 % glycerol solution (0.15 ml) and then stored at −80 °C.

### Preparation of *E. coli* strains

*Caulobacter crescentus xylX, xylA*, *xylB*, *xylC* and *xylD* (*xylX_Cc_ xylA_Cc_*, *xylB_Cc_*, *xylC_Cc_*, *xylD_Cc_*; Genbank [23] gene ID 7329906, 7329905, 7329904, 7329903 and 7329902, respectively) were optimized for expression in *E. coli* (Fig. S2), synthesized by Biomatik and inserted into pET-20b(+) using the *NdeI* and *NotI* restriction sites to obtain inducible expression. *E. coli* BL21(DE3) pLysS pET-20b(+)*xylX_Cc_,*, pET-20b(+)*xylA_Cc_*, pET-20b(+)*xylB_Cc_*, pET-20b(+)*xylC_Cc_* and pET-20b(+)*xylD_Cc_* were prepared by transforming *E. coli* BL21(DE3) pLysS by heat shock. Transformants were selected on LB-Cb-Cm agar supplemented with glucose. *E. coli* BL21(DE3) pLysS was also transformed with empty pET-20b(+).

The host strains for constitutive expression, *E. coli* BW25113 *Δicd ΔxylAB *:: Cm^R^ and *E. coli* BW25113 *ΔxylAB* :: Cm^R^, were constructed [24] by deleting *icd* in the former strain, and then deleting *E. coli xylA* and *xylB* (*xylA_Ec_* and *xylB_Ec_*) in a single step, since the two genes are adjacent on the chromosome. A chloramphenicol resistance cassette was left at the *ΔxylAB* site. The primers used for *icd* deletion were 5′-ATATG CAACGTGGTGGCAGACGAGCAAACCAGTAGCGCTCG AAGGAGAGGGTGTAGGCTGGAGCTGCTTC-3′ and 5′-AAAACAACGGGAGCGTTACGCTCCCGTTAATAAATT TAACAAACTACGGCATGGGAATTAGCCATGGTCC-3′. The primers used for *xylAB_Ec_* deletion were 5′-CGACATCATCCATCACCCGCGGCATTACCTGATTATGGAGTTCAATATGGTGTAGGCTGGAGCTGCTTC-3′ and 5′-CCC GGTCAGGCAGGGGATAACGTTTACGCCATTAATGGC AGAAGATGGGAATTAGCCATGGTCC-3′. The deletions were confirmed by PCR [24].

*E. coli* BW25113 *Δicd ΔxylAB *:: Cm^R^ was transformed by electroporation (Gene Pulser Xcell System; Bio-Rad) with six different plasmid libraries containing combinations of *xylX_Cc_*, *xylA_Cc_* and *xylB_Cc_*, and combinations of four different promoters (pL, pAspC, pR and pOSMY; Fig. S3). The plasmid backbone, pCX, contained a pMB1 origin of replication and a kanamycin resistance cassette. Libraries were generated using Ingenza Ltd’s proprietary combinatorial DNA assembly method (inABLE), where the required genes and promoters are left with overhangs using Type IIS restriction enzymes and then ligated with specific linkers. *E. coli* BW25113 *Δicd ΔxylAB *:: Cm^R^ and *E. coli* BW25113 *ΔxylAB *:: Cm^R^ were also transformed with the pCX and pCL empty vectors, and pCL-*xylCD_Cc_*. The pCL plasmid and pCL-*xylCD_Cc_* were also prepared using inABLE and contained a p15A origin of replication, an ampicillin resistance cassette and a pL promoter. Transformants were selected on LB-Cb or LB-Km agar, and cells from the pCX libraries were pooled for screening by resuspending the colonies in LB (2 ml) using a spreader, and mixing the cell suspension (0.85 ml) with 80 % glycerol solution (0.15 ml) for storage at −80 °C.

### *In vitro* enzyme activity assays

Enzyme activity was confirmed by measuring the conversion of d-xylose to 2-oxoglutarate using mixtures of the individual cell-free extracts. *E. coli* BL21(DE3) pLysS pET-20b(+)*xylX_Cc_,*, pET-20b(+)*xylA_Cc_*, pET-20b(+)*xylB_Cc_*, pET-20b(+)*xylC_Cc_* and pET-20b(+)*xylD_Cc_* were plated onto LB-Cb-Cm agar from frozen stock cultures, and single colonies were inoculated into LB-Cb-Cm (20 ml). After overnight growth, these precultures were used to inoculate LB-Cb-Cm (100 ml) to an optical density (OD_600_) of approximately 0.1. Enzyme expression was induced at an OD_600_ of approximately 0.6, by adding IPTG (0.4 mM), decreasing the temperature as specified in the text. The cells were collected 14–18 h after induction by centrifugation at 15 000 ***g*** for 10 min, resuspended in cold 100 mM Tris-HCl buffer, pH 7.4, and lysed with a Constant Systems cell disruptor, with all procedures being performed at 4 °C. Cell debris was removed by centrifugation at 15 000 ***g*** for 15 min. Enzyme expression was checked by SDS-PAGE using a Mini-Protean electrophoresis system and TGX precast gels stained with QC colloidal Coomassie protein stain. Total protein concentration was determined using the Bio-Rad DC protein assay kit. Cell-free extracts from the *E. coli* strains expressing individual *C. crescentus* genes encoding for XDH, XL, XD, KdxD and DPDH were mixed together in the same buffer and assayed upon the addition of MgCl_2_, d-xylose or d-KDX, NAD and NADP, using the concentrations and conditions specified in the text. The reaction mixtures were analysed after 6 h.

### *In vivo* screening for a functional Weimberg pathway

Frozen stock cultures of *E. coli* BW25113 *Δicd ΔxylAB *:: Cm^R^ strains transformed with the pCX plasmid libraries (100 µl) were washed three times by centrifugation at 15 000 ***g*** for 5 min, resuspended in sterilized phosphate-buffered saline (PBS; pH 7.4) (containing NaCl, 8 g l^−1^; KCl, 0.2 g l^−1^; Na_2_HPO_4_, 1.42 g l^−1^; KH_2_PO_4_, 0.24 g l^−1^) and plated onto ML-XKm medium, alongside *E. coli* BW25113 and *E. coli* BW25113 *Δicd ΔxylAB *:: Cm^R^ containing pCX as controls. After incubation, 12 single colonies from different libraries were randomly used to inoculate a 100-well Bioscreen plate containing ML-XKm medium (360 µl culture volume). Growth was measured at 37 °C with shaking using a Bioscreen C (Labsystems, Helsinki, Finland), which automatically measures OD_600_. Growth rates were calculated by selecting data points from the exponential phase by visual inspection of a plot of *ln*OD_600_ versus time and using linear regression. The fastest growing strains (25) amongst all the libraries were isolated on LB-Km agar and their growth rates were measured as above by preculturing them in ML-XKm (20 ml) and using the precultures to inoculate well-plate cultures at an initial OD_600_ of 0.1. The three strains with the fastest growth rates were reisolated as above, and the plasmids were extracted using a QIAprep Spin Midiprep kit and sent to Eurofins for sequencing.

The growth rates of the isolated strains were also measured accurately by using single colonies grown on ML-XKm agar plates to inoculate precultures in ML-XKm medium (20 ml). After ~24 h, the cells were collected by centrifugation at 10 000 ***g*** for 20 min, washed three times aseptically in sterilized PBS and used to inoculate ML-XKm or ML-GKm medium (100 ml). The OD_600_ was monitored at intervals and the growth rates were calculated as described above. When required, samples (1 ml) were also taken at intervals to analyse the concentration of metabolites.

### Analytical methods

The OD_600_ of cultures was determined by aseptically sampling growing cultures, diluting in water to OD<0.8 and measuring the OD at 600 nm. The dry cell weight was estimated using a calibration curve of OD_600_ versus dry weight. Biomass yields were calculated by dividing the total biomass produced (*g*_dry weight_) by the total amount of d-xylose consumed (*g*). d-Xylose and all of the metabolites were quantified by high-pressure liquid chromatography (HPLC) using an Agilent 1200 Series HPLC system. Samples from cultures or *in vitro* enzyme assays were centrifuged and filtered (0.2 µm filters), and filtrate samples (20 µl) injected onto a Rezex ROA-Organic Acid H+(8 %) column (Phenomenex) at 55 °C using an autosampler. The mobile phase was 0.005 M H_2_SO_4_ (0.5 ml min^−1^). The sample components were detected using a refractive index detector (G1362A Detector) at 35 °C and a UV detector (G1314B Variable Wavelength Detector) at 215 nm. Data analysis was performed with ChemStation software, using calibration curves prepared using authentic standards of each compound (0.1–50 mM).

d-Xylonate and d-xylonolactone co-elute on HPLC, and, therefore were detected and quantified using the hydroxamate method described by Lien [25]. This method converts d-xylonate to d-xylonolactone and, for this reason, the text refers to the two compounds as d-xylonate/d-xylonolactone. This assay was sensitive to 0.1 g l^−1^ d-xylonolactone.

## Results

### *In vitro* demonstration of the Weimberg pathway in *E. coli*

To ensure the successful engineering of the constitutive Weimberg pathway, we confirmed that the *C. crescentus* genes, *xylXAB_Cc_*, encoding for KdxD, DPDH and XDH, respectively, could be expressed in *E. coli* in active form. The native *E. coli* genes, *yjhG* and *yagF*, encode XD activity [26–29], and d-xylonate can be formed by spontaneous chemical hydrolysis of d-xylonolactone [30], so the expression of *xylXAB_Cc_* alone ought to be sufficient to obtain a functional Weimberg pathway in *E. coli*.

The codon-optimized synthetic genes were inserted individually into the pET-20b(+) vector, and the resulting plasmids were transformed into *E. coli* BL21(DE3) pLysS to produce *E. coli* BL21(DE3) pLysS pET-20b(+)*xylX_Cc,_* pET-20b(+)*xylA_Cc_* and pET-20b(+)*xylB_Cc_*. XDH and KdxD (encoded by x*ylB_Cc_* and *xylX_Cc_*, respectively) were expressed as soluble enzymes when the cells were grown at 37 °C and the temperature was decreased to 30 °C after induction, but DPDH (encoded by *xylA_Cc_*) was expressed most efficiently when the temperature was decreased from 37 to 18 °C after induction (Fig. S5).

**Fig. 2.**
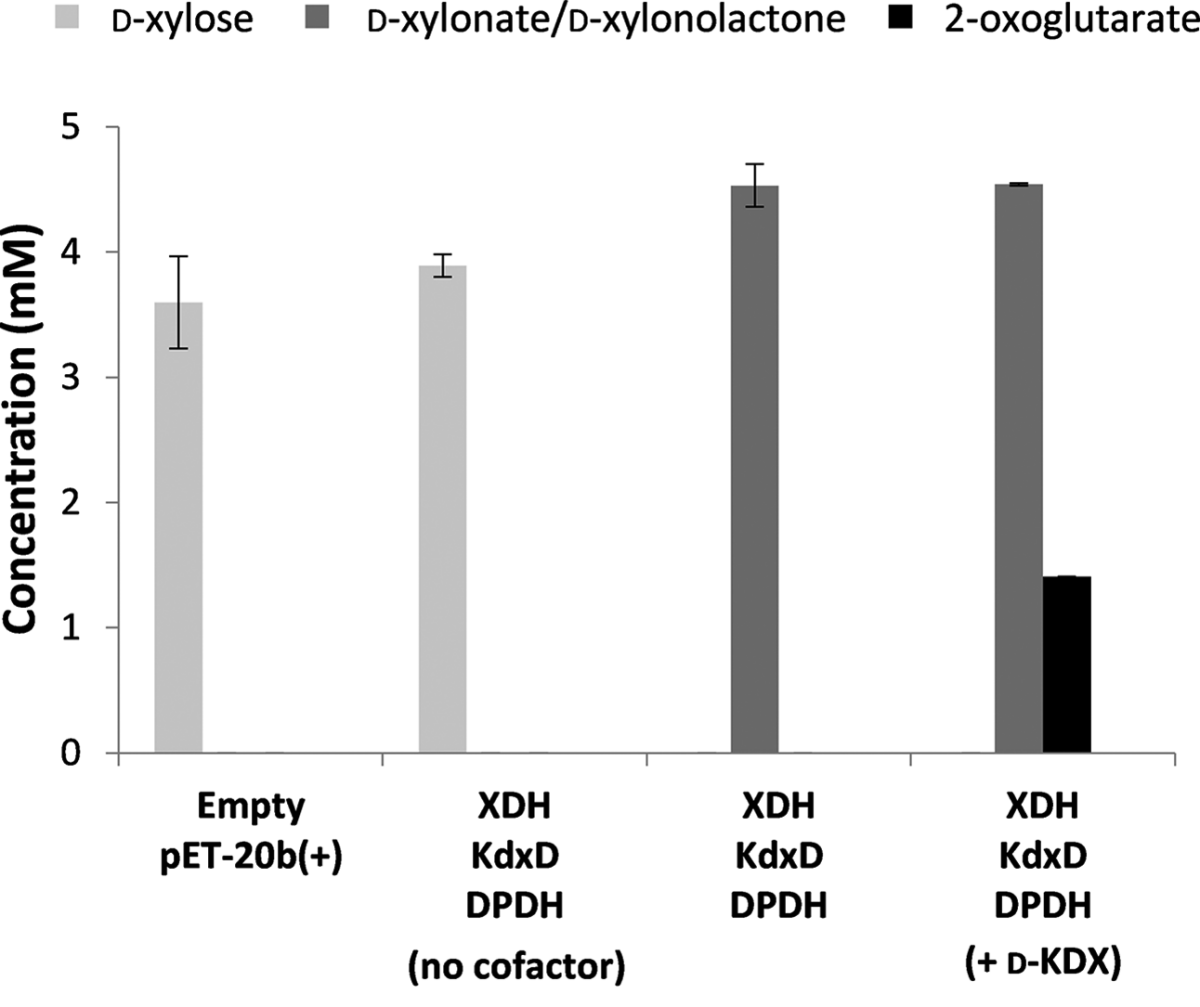
*In vitro* characterization of the Weimberg pathway. Cell-free extracts of *E. coli* BL21(DE3) pLysS pET-20b(+)*xylB_Cc,_* pET-20b(+)*xylX_Cc_* and pET-20b(+)*xylA_Cc_*, expressing XDH, KdxD or DPDH, respectively, were obtained individually, mixed as indicated and assayed for the formation of 2-oxoglutarate from d-xylose (5 mM) in reaction mixtures (1 ml in microcentrifuge tubes) containing 100 mM Tris-HCl buffer, pH 7.4, MgCl_2_ (50 mM), NAD^+^ (10 mM) and NADP^+^ (10 mM) at 37 °C, with shaking at 200 r.p.m. The amount of total protein added from each cell-free extract was 0.1 mg. Where indicated, d-KDX (2 mM) was also added to the reaction mixture in addition to d-xylose. The controls consisted of cell-free extract from *E. coli* BL21 (DE3) pLysS pET-20b(+) and a cofactor-free control containing XDH, KdxD and DPDH. Product concentrations were determined after 6 h. d-KDX could not be detected in any of the reactions. Assays were performed in triplicate, and the means and standard errors are shown.

The activity of XDH, KdxD and DPDH was tested by mixing cell-free extracts from *E. coli* BL21(DE3) pLysS pET-20b(+)*xylX_Cc,_* pET-20b(+)*xylA_Cc_* and pET-20b(+)*xylB_Cc_* and testing for the conversion of d-xylose to 2-oxoglutarate in the presence of NAD and NADP, which are required for XDH and DPDH activity, respectively (Fig. 2). Control reactions without cofactors, and an extract of an isogenic strain transformed with the empty-pET-20b(+) vector [*E. coli* BL21(DE3) pLysS pET-20b(+)] consumed 28±7 and 23±1 % of the d-xylose, respectively, presumably due to the native PPP in the host strain, but no metabolic products from the Weimberg pathway could be detected, as expected.

By contrast, d-xylose was consumed completely by the mixture of cell-free extracts containing XDH, KdxD and DPDH. However, d-xylonolactone/d-xylonate accumulated (4.53±0.17 mM from 5 mM d-xylose; Fig. 2), and neither d-KDX nor 2-oxoglutarate could be detected. This indicated that XDH was active, and was able to convert d-xylose to d-xylonolactone, but that there was a bottleneck further along the pathway.

To identify this bottleneck, d-KDX was added to the xylose-containing reaction mixture. This resulted in the formation of 2-oxoglutarate in addition to d-xylonolactone/*D*-xylonate (Fig. 2), and, thus demonstrated that the downstream pathway enzymes, KdxD and DPDH, were active. This suggests that the XD activity provided by the native *E. coli* enzymes encoded by *yjhG* and *yagF* was insufficient to allow oxidation of d-xylonate to d-KDX in these enzyme assays *in vitro*, even though these activities are known to be sufficient to support *in vivo* oxidation of d-xylose [26]. Thus, we have confirmed that the Weimberg pathway can function *in vitro*, but that conversion of d-xylonate to d-KDX may be a limiting step in the pathway, as proposed previously [18].

### *In vivo* optimization of constitutive expression of the Weimberg pathway for growth of *E. coli* on d-xylose

The next step was to engineer a strain that could grow constitutively on d-xylose using the Weimberg pathway. A combinatorial approach was used to optimize pathway expression, by systematically testing different constitutive promoters and synthetic operons with the pathway genes in different orders.

The pathway functionality was tested by employing a growth-based screen, using an auxotrophic mutant of *E. coli* BW25113 that requires 2-oxoglutarate for growth, by knocking out the isocitrate dehydrogenase gene, *icd*. In *E. coli*, this enzyme is essential for production of 2-oxoglutarate *de novo*. Since 2-oxoglutarate is crucial for cell growth and viability, this made the cells dependent on the efficient expression of the Weimberg pathway to convert *d*-xylose to 2-oxoglutarate. We also deleted *xylA_Ec_* and *xylB_Ec_*, encoding xylose isomerase and xylulokinase, respectively, thus inactivating the first two steps of the PPP (Fig. 1). Therefore, the resulting strain, *E. coli* BW25113 *Δicd ΔxylAB *:: Cm^R^, could be used as the host to screen for an active Weimberg pathway.

Six different combinatorial plasmid libraries were generated using *xylX_Cc_*, *xylA_Cc_* and *xylB_Cc_*, encoding KdxD, DPDH and XDH, respectively. These three genes alone should be sufficient for d*-*xylose oxidation *via* the Weimberg pathway in growing *E. coli* cells [26–29]. The six libraries employed the same plasmid backbone (pCX), and contained a synthetic operon with the *xylX_Cc_*, *xylA_Cc_* and *xylB_Cc_* genes in different orders, under the control of four different known constitutive promoters (pL, pAspC, pR and pOSMY; Fig. S3; Table 1).

**Table 1.** Summary of the design of the plasmid libraries for the constitutive expression of *xylX_Cc_*, *xylA_Cc_* and *xylB_Cc_*

Library #	Order of genes in synthetic operon	Promoters
1	*xylB*XA*_Cc_*	Four possible promoters pL pAspC pR pOSMY
2	*xylB*AX*_Cc_*	
3	*xylX*AB*_Cc_*	
4	*xylX*BA*_Cc_*	
5	*xylA*BX*_Cc_*	
6	*xylA*XB*_Cc_*	

*E. coli* BW25113 *Δicd ΔxylAB *:: Cm^R^ was transformed with the plasmid libraries. The variants were then screened by plating onto ML minimal medium containing d-xylose as sole carbon source and kanamycin (ML-XKm), and compared with the control strains with empty pCX, *E. coli* BW25113 pCX and *E. coli* BW25113 *Δicd ΔxylAB *:: Cm*^R^* pCX.

*E. coli* BW25113 *Δicd ΔxylAB *:: Cm*^R^* pCX did not grow at all, due to lack of the PPP and isocitrate dehydrogenase, whereas wild-type *E. coli* BW25113 pCX produced colonies after 14–16 h (Fig. S6), due to the presence of the PPP. Colonies were also visible on the plates containing *E. coli* BW25113 *Δicd ΔxylAB *:: Cm*^R^* transformed with the plasmid libraries after 24–48 h (Fig. S6). This demonstrated that the expression of KdxD, DPDH and XDH allowed *E. coli* to grow on d-xylose by using the Weimberg pathway, and confirmed that the native XD activity and the chemical hydrolysis of xylonolactone were sufficient to support growth and 2-oxoglutarate production [26].

**Fig. 3.**
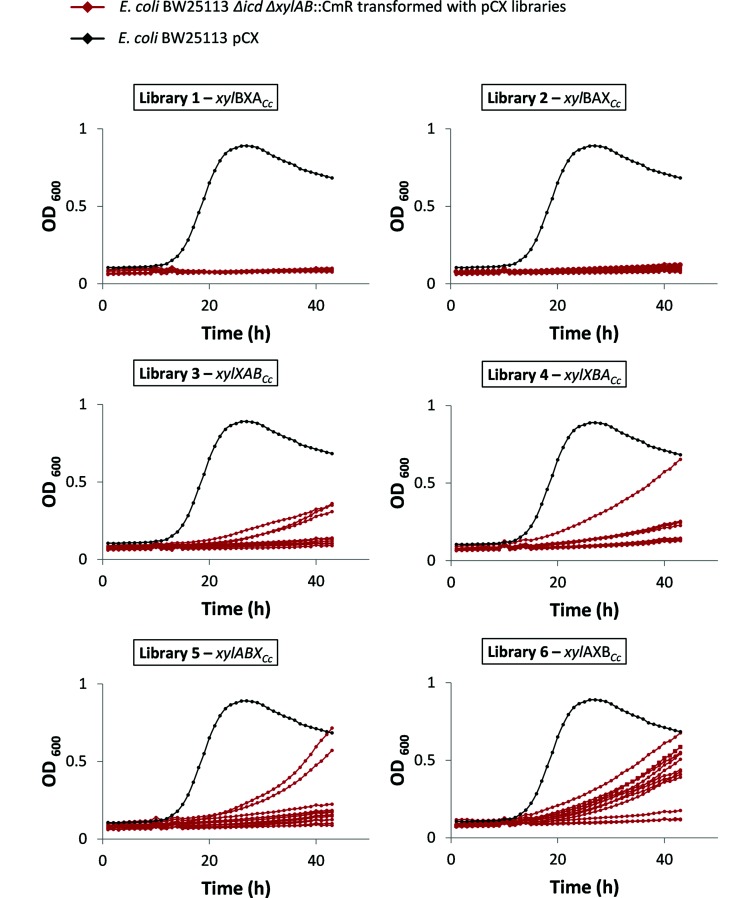
Growth in microtitre plates of strains isolated from Weimberg pathway libraries. *E. coli* BW25113 *Δicd ΔxylAB *:: Cm^R^ transformed with pCX libraries were selected for growth on d-xylose-containing agar plates, and 12 single colonies were picked and inoculated into Bioscreen microtitre plates containing ML-XKm (360 µl total volume per well). Growth was followed at 37 °C with shaking (red lines). *E. coli* BW25113 pCX was also analysed as a control (black lines). The figure shows the growth of colonies from libraries 1 to 6 in separate graphs, and the order of the *C. crescentus* genes (*xylA_Cc_*, *xylB_Cc_* and *xylX_Cc_*) in the synthetic operon is indicated.

The incubation time required to obtain visible colonies varied amongst the six libraries, and the size of the colonies varied within each library, indicating that the order of the genes and the promoters influenced the growth efficiency. Twelve strains from each library were selected at random, isolated and tested for growth on d-xylose by inoculating single colonies into a microtitre plate, and compared with wild-type *E. coli* BW25113 pCX (Fig. 3). All of the variants grew on d-xylose, although more slowly than *E. coli* BW25113 pCX using the native PPP. However, the growth rates varied significantly, both within the same library and amongst different libraries. When *xylX_Cc_* (encoding KdxD) or *xylA_Cc_* (encoding DPDH) were at the first position in the synthetic operon (libraries 3, 4, 5 and 6), there was a broad distribution of different growth rates amongst the individual strains, whereas all the strains grew very slowly when *xylB_Cc_* (encoding XDH) was the first gene (libraries 1 and 2).

A further round of screening was then performed, in which the fastest growing strains amongst libraries 3, 4, 5 and 6–25 in total – were retested for growth on d-xylose in microtitre plates, but this time by inoculation from precultures to ensure that all of the cultures had the same initial OD_600_ (data not shown). The three variants with the highest growth rates were isolated (two from library 5 and one from library 6), and the plasmids extracted and sequenced. The two variants from library 5 contained the same plasmid, pCX-pL-*xylABX_Cc_*, whilst the library 6 variant contained pCX-pL-*xylAXB_Cc_.* Therefore, the pL promoter with *xylA_Cc_* at the first position in the operon provided the most beneficial combination for efficient growth using the Weimberg pathway.

The growth rates of *E. coli* BW25113 *Δicd ΔxylAB *:: Cm^R^ pCX-pL-*xylABX_Cc_* and *E. coli* BW25113 *Δicd ΔxylAB *:: Cm^R^ pCX-pL-*xylAXB_Cc_* were measured accurately in shake flask cultures (Fig. S7). *E. coli* BW25113 *Δicd ΔxylAB *:: Cm^R^ pCX-pL-*xylAXB_Cc_* grew at a rate of 0.20±0.01 h^−1^, whilst *E. coli* BW25113 *Δicd ΔxylAB *:: Cm^R^ pCX-pL-*xylABX_Cc_* grew at a rate of 0.18±0.01 h^−1^, 42 and 38 %, respectively, of the growth rate of *E. coli* BW25113 pCX, which utilizes d-xylose *via* the PPP (0.48±0.01 h^−1^). Neither of the engineered strains was able to grow on glucose as sole carbon source (Fig. S7), due to the deletion of *icd*, which confirms that the Weimberg pathway is required for the production of 2-oxoglutarate. Due to the faster growth rate, *E. coli* BW25113 *Δicd ΔxylAB *:: Cm^R^ pCX-pL-*xylAXB_Cc_* was selected for further experiments

### Strain optimization

Although *E. coli* BW25113 *Δicd ΔxylAB *:: Cm^R^ pCX-pL-*xylAXB_Cc_* was able to grow constitutively on d-xylose using the Weimberg pathway, the growth rate was slower than that for *E. coli* BW25113 pCX, which utilizes the PPP. However, the enzyme assays had indicated that spontaneous hydrolysis of d-xylonolactone and the native *E. coli* XD activity encoded by *yjhG* and *yagF* could be rate-limiting for the complete oxidation of xylose to 2-oxoglutarate *in vitro* (Fig. 2). Therefore, we tested the addition of XL and XD, encoded by *xylC_Cc_* and *xylD_Cc_*, to increase the rate of d-xylonate formation from d-xylonolactone [31, 32] and increase the rate of d-KDX formation from d-xylonate [18].

**Fig. 4.**
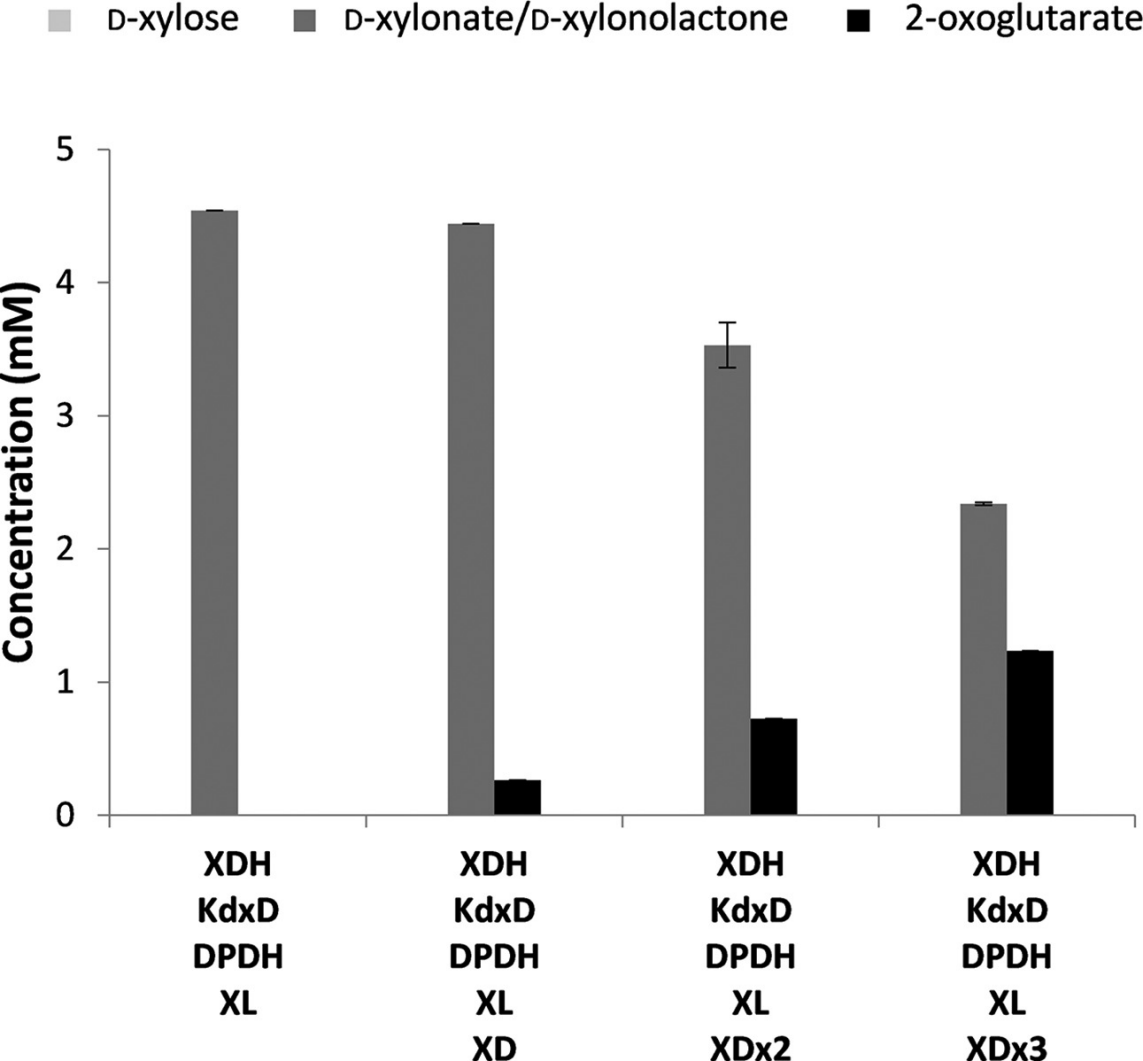
*In vitro* effect of addition of *C. crescentus* XL and XD. Cell-free extracts of *E. coli* BL21(DE3) pLysS pET-20b(+)*xylB_Cc,_* pET-20b(+)*xylX_Cc_*, pET-20b(+)*xylA_Cc_*, pET-20b(+)*xylC_Cc_* and pET-20b(+)*xylD_Cc_*, expressing XDH, KdxD, DPDH, XL or XD, were obtained individually, mixed as indicated and assayed for the formation of 2-oxoglutarate from d-xylose (5 mM) in reaction mixtures (1 ml in microcentrifuge tubes) containing 100 mM Tris-HCl buffer, pH 7.4, MgCl_2_ (50 mM), NAD^+^ (10 mM) and NADP^+^ (10 mM) at 37 °C, with shaking at 200 r.p.m. The amount of total protein added from each cell-free extract was 0.1 mg. Where indicated, the amount of XD was increased to 0.2 mg (XDx2) or 0.3 mg (XDx3). Product concentrations were determined after 6 h. d-KDX could not be detected in any reactions. Assays were performed in triplicate, and the means and standard errors are shown.

Initially, we confirmed that *xylC_Cc_* and *xylD_Cc_* could be expressed in *E. coli*. XL and XD were expressed as a soluble proteins when *E. coli* BL21(DE3) pLysS pET-20b(+)*xylC_Cc_* and *E. coli* BL21(DE3) pLysS pET-20b(+)*xylD_Cc_*, respectively, were grown at 37 °C and cooled to 30 and 18 °C after induction, respectively (Fig. S5). Enzyme activity was confirmed by demonstrating that d-xylose was fully consumed and converted to 2-oxoglutarate *in vitro*, using mixtures of cell-free extracts from *E. coli* BL21(DE3) pLysS pET-20b(+)*xylC_Cc_* and *E. coli* BL21(DE3) pLysS pET-20b(+)*xylD_Cc_*, expressing *C. crescentus* XL and XD, respectively, with extracts containing XDH, KdxD and DPDH. 2-Oxoglutarate was not produced when only XL, XDH, KdxD and DPDH were present (Fig. 4). Therefore, XD activity was the limiting step of the pathway *in vitro*. However, the XD-containing enzyme mixture only produced 0.26±0.03 mM 2-oxoglutarate, and d-xylonolactone/d-xylonate accumulation was still observed (4.44±0.17 mM). Therefore, the concentration of XD was doubled and tripled, and this resulted in increased formation of 2-oxoglutarate (0.72±0.03 and 1.24±0.05 mM, respectively) and decreased accumulation of d-xylonolactone/d-xylonate (3.53±0.05 and 2.34±0.05 mM, respectively), further confirming that XD activity is the limiting step in 2-oxoglutarate formation *in vitro*.

**Fig. 5.**
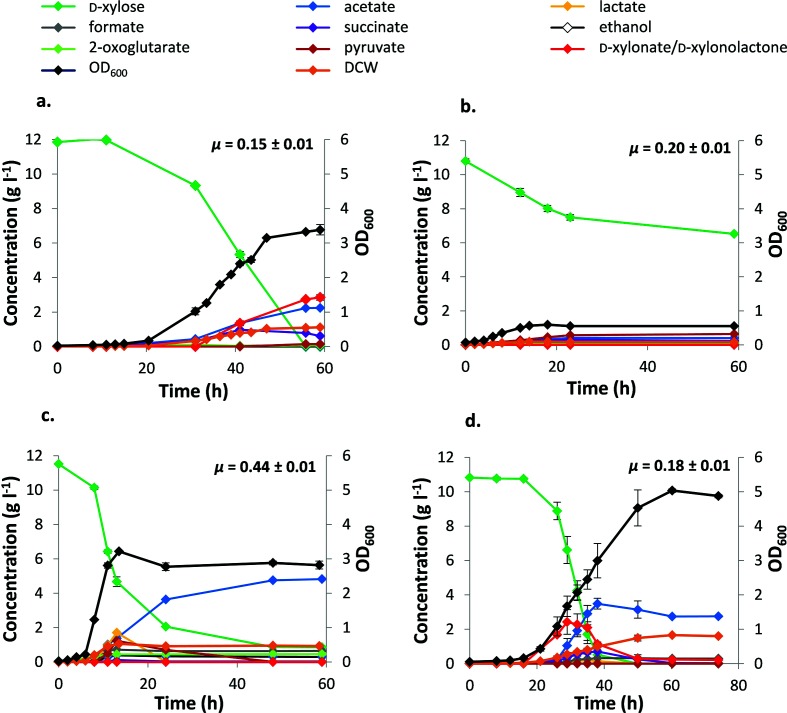
Analysis of growth and metabolic intermediates. Growth and metabolic profile of *E. coli* BW25113 *Δicd ΔxylAB *:: Cm^R^ pCX-pL-*xylAXB_Cc_* pCL (a), *E. coli* BW25113 *Δicd ΔxylAB *:: Cm^R^ pCX-pL-*xylAXB_Cc_* pCL-*xylCD_Cc_* (b), *E. coli* BW25113 pCX pCL (c) and *E. coli* BW25113 *ΔxylAB *:: Cm^R^ pCX-pL-*xylAXB_Cc_* pCL (d) when grown on ML-XKm-Cb. The cells were grown in flasks (100 ml culture volume) and the dry cell weight (DCW) and OD_600_ are indicated. The presence of d-xylose, acetate, lactate, formate, succinate, ethanol, 2-oxoglutarate, pyruvate, d-KDX and d-xylonolactone/d-xylonate was analysed at intervals. Assays were performed in triplicate, and the means and standard errors are shown. The growth rate for each strain (*µ*) is also indicated.

We then tested the effect of expressing *C. crescentus* XD and XL on the growth of *E. coli* on d*-*xylose *in vivo* by transforming *E. coli* BW25113 *Δicd ΔxylAB *:: Cm^R^ pCX-pL-*xylAXB_Cc_* with an extra plasmid, pCL-*xylCD_Cc_*, under the control of the pL promoter to produce the complete Weimberg pathway from *C. crescentus.* Although *E. coli* BW25113 *Δicd ΔxylAB *:: Cm^R^ pCX-pL-*xylAXB_Cc_* pCL-*xylCD_Cc_* grew on d*-*xylose at a rate of 0.20±0.01 h^−1^, the cells stopped growing after they reached a dry cell weight (DCW) of only 0.18±0.01 g l^−1^ (OD_600_ of 0.59±0.01; Fig. 5b; Table 2). By contrast *E. coli* BW25113 *Δicd ΔxylAB *:: Cm^R^ pCX-pL-*xylAXB_Cc_* grew on d*-*xylose at exactly the same rate, but the final DCW was 1.03±0.02 g l^−1^ (OD_600_ of 3.12±0.01; Fig. S7). Therefore, the presence of pCL-*xylCD_Cc_* caused premature cessation of growth, but had no effect on the growth rate.

**Table 2.** Summary of growth rate, final biomass and metabolite data. The full values for the metabolite data are available in Table S1

**Strain**	**Growth rate (µ^−1^)**	**Final biomass** **Dry cell weight (g l^−1^) (OD_600_)**	**Key metabolite data (g l^−1^)**
*E. coli* BW25113 pCX pCL (Fig. 5c)	0.44±0.01	1.06±0.01 (3.22±0.01)	*Final concentration:* d-xylose (0.86±0.01) Acetate (4.80±0.04) Formate (0.65±0.01) Ethanol (0.29±0.01) Succinate (0.01±0.01) 2-oxoglutarate (0.01±0.01) Pyruvate (0.01±0.01) Lactate (0.01±0.01) *Transient accumulation:* Lactate (1.71±0.03) Pyruvate (1.23±0.03) Formate (0.72±0.03) Succinate (0.08±0.01) 2-oxoglutarate (0.45±0.01)
*E. coli* BW25113 *Δicd ΔxylAB *:: Cm^R^ pCX-pL-*xylAXB_Cc_* pCL (Fig. 5a)	0.15±0.01	1.12±0.05 (3.38±0.14)	*Final concentration:* d-xylonolactone/d-xylonate (2.85±0.16) Acetate (2.25±0.01) Succinate (0.61±0.03) Pyruvate (0.14±0.01) Formate (0.07±0.01) 2-oxoglutarate (0.06±0.01) *Transient accumulation:* Succinate (0.98±0.07) 2-oxoglutarate (0.11±0.01)
*E. coli* BW25113 *ΔxylAB *:: Cm^R^ pCX-pL-*xylAXB_Cc_* pCL (Fig. 5d)	0.18±0.01	1.66±0.02 (5.04±0.05)	*Final concentration:* Acetate (2.74±0.06) Formate (0.30±0.01) d-xylonolactone/d-xylonate (0.21±0.02) Succinate (0.03±0.01) *Transient accumulation:* Acetate (3.49±0.31) d-xylonolactone/d-xylonate (2.43±0.71) Succinate (0.71±0.01) Lactate (0.20±0.01)
*E. coli* BW25113 *Δicd ΔxylAB *:: Cm^R^ pCX-pL-*xylAXB_Cc_* pCL-*xylCD_Cc_* (Fig. 5b)	0.20±0.01	0.18±0.01 (0.59±0.01)	*Final concentration:* d-xylose (6.52±0.05) Pyruvate (0.64±0.02) Acetate (0.41±0.01) Succinate (0.23±0.04) Lactate (0.16±0.04) 2-oxoglutarate (0.11±0.00) Formate (0.08±0.01)
*E. coli* BW25113 *ΔxylAB *:: Cm^R^ pCX-pL-*xylAXB_Cc_* pCL-*xylCD_Cc_*	0.20±0.01	0.10±0.01 (0.31±0.01)	*Final concentration:* d-xylose (6.86±0.06) Pyruvate (0.40±0.02) Acetate (0.44±0.01) Succinate (0.24±0.02) Lactate (0.23±0.02) Formate (0.06±0.03)

We tested the hypothesis that the cessation of growth might be due to the presence of the additional pCL plasmid by comparing the growth of an isogenic strain transformed with the empty pCL vector. *E. coli* BW25113 *Δicd ΔxylAB *:: Cm^R^ pCX-pL-*xylAXB_Cc_* pCL reached a final DCW of 1.12±0.05 g l^−1^ (OD_600_ of 3.38±0.14), similar to the progenitor strain, but the growth rate was 25 % lower (0.15±0.01 h^−1^; Fig. 5a; Table 2). Thus, the presence of the pCL empty vector only affected the growth rate but not the biomass yield, and the cessation of growth observed with *E. coli* BW25113 *Δicd ΔxylAB *:: Cm^R^ pCX-pL-*xylAXB_Cc_* pCL-*xylCD_Cc_* was due to expression of *XylC_Cc_* (encoding XL) and/or *xylD_Cc_* (encoding XD). Rather than improving the ability of the strain to use d-xylose *via* the Weimberg pathway, the presence of *C. crescentus* XD and XL was detrimental. Therefore, further strain optimization was focused on improving the growth of strains expressing *xylAXB_Cc_*, and relying upon spontaneous hydrolysis of xylonolactone and the native *E. coli* XD activity to produce d-KDX.

*E. coli* BW25113 *Δicd ΔxylAB *:: Cm^R^ pCX-pL-*xylAXB_Cc_* pCL-*xylCD_Cc_* contained the *icd* deletion, which had been introduced to enable rapid strain selection. Isocitrate dehydrogenase is required for operation of the complete oxidative TCA cycle, and the *Δicd* mutants would instead be reliant on alternative pathways for oxidation of the 2-oxoglutarate formed by the Weimberg pathway (e.g. the glyoxylate shunt or the succinate oxidation pathway). Such pathways generate much less energy than the TCA cycle, which may account for the observed reduction in growth rate. Therefore, we tested the effect of restoring the native isocitrate dehydrogenase activity by preparing isogenic strains lacking the *icd* deletion.

*E. coli* BW25113 *ΔxylAB *:: Cm^R^ pCX-pL-*xylAXB_Cc_* pCL-*xylCD_Cc_* behaved in almost exactly the same way as the *Δicd* counterpart, with exactly the same growth rate and with growth stopping prematurely at a DCW of 0.10±0.01 g l^−1^ (OD_600_ of 0.31±0.01; Table 2). Therefore, the growth inhibition caused by *xylCD_Cc_* expression cannot be overcome by restoring a fully functional TCA cycle.

By contrast, *E. coli* BW25113 *ΔxylAB *:: Cm^R^ pCX-pL-*xylAXB_Cc_* pCL was able to grow 20 % faster than the *Δicd* counterpart (0.18±0.01 h^−1^), and reached a final biomass concentration that was 48 % higher (DCW of 1.66±0.02 g l^−1^ and OD_600_ of 5.04±0.05), so that the growth yield on d-xylose was 60 % higher (Fig. 5d; Table 2). The final biomass concentration was also 57 % higher than that of the PPP-dependent strain, *E. coli* BW25113 pCX pCL, and the growth yield had increased by 53.5 % (Fig. 5; Table 2), although the growth rate was still 59 % lower (Fig. 5; Table 2) than that for *E. coli* BW25113 pCX pCL.

### Formation of intermediates and metabolic products

To further characterize the newly developed strains, we also monitored accumulation of intermediates and fermentation products during the growth of each of the strains (Table 2; Fig. 5). *E. coli* BW25113 pCX pCL showed rapid consumption of d-xylose *via* the PPP, followed by slower consumption after growth stopped. There was transient accumulation of pyruvate, lactate, succinate, formate and 2-oxoglutarate on the approach to the stationary phase, followed by accumulation of ethanol, formate and acetate. The accumulation of these fermentation products was likely due to oxygen limitation, which is known to occur in shake flasks [33], and this, together with the accumulation of these inhibitory organic acids [34–36], may explain why growth stopped before d-xylose had been completely consumed.

By contrast, *E. coli* BW25113 *Δicd ΔxylAB *:: Cm^R^ pCX-pL-*xylAXB_Cc_* pCL, with the *xylAXB_Cc_* genes and the *icd* deletion, was able to consume the d-xylose completely, but d-xylonolactone/d-xylonate began to accumulate after 41 h. This is consistent with the experiments *in vitro*, which had suggested that the formation of d-KDX from d-xylonolactone/d-xylonate was a rate-limiting step (Fig. 4). This organism also produced acetate, but to a final concentration that was 47 % lower than in the cultures of *E. coli* BW25113 pCX pCL, although both strains accumulated 2-oxoglutarate transiently, followed by later reconsumption. Formate was present in trace amounts, ethanol was not observed, and the transient accumulation of lactate was absent. The limited ability to consume d-xylonolactone/*D*-xylonate suggests that the growth of *E. coli* BW25113 *Δicd ΔxylAB *:: Cm^R^ pCX-pL-*xylAXB_Cc_* pCL was limited by incomplete metabolism of d-xylose, rather than being limited by the accumulation of inhibitory organic acids.

Interestingly, succinate and pyruvate also accumulated. This may be due to the *icd* deletion, which prevents the operation of the TCA cycle, potentially affecting the metabolism of these intermediates. Indeed, the isogenic strain with restored isocitrate dehydrogenase activity (*E. coli* BW25113 *ΔxylAB *:: Cm^R^ pCX-pL-*xylAXB_Cc_* pCL) produced acetate and formate, but no pyruvate; succinate was produced transiently, but at a much lower concentration compared to that for *E. coli* BW25113 *Δicd ΔxylAB *:: Cm^R^ pCX-pL-*xylAXB_Cc_* pCL. Furthermore, d-xylonolactone/d-xylonate accumulation was also transient, and was consumed almost completely thereafter, alongside consumption of d-xylose. Once the d-xylose had been fully consumed, slow growth continued, and the accumulated d-xylonolactone/d-xylonate and succinate continued to be consumed. When growth stopped, the main detectable metabolic products were d-xylonolactone/*D*-xylonate, acetate and formate, at lower accumulation compared to all of the other strains tested. This indicates that d-xylose oxidation was more efficient, and may explain why the strain was able to reach a higher final biomass and growth yield than *E. coli* BW25113 *Δicd ΔxylAB *:: Cm^R^ pCX-pL-*xylAXB_Cc_* pCL and *E. coli* BW25113 pCX pCL (Table 2).

The presence of the *xylCD_Cc_* genes had a detrimental effect on d-xylose utilization, whether or not the *icd* deletion was present. Thus, *E. coli* BW25113 *Δicd ΔxylAB *:: Cm^R^ pCX-pL-*xylAXB_Cc_* pCL-*xylCD_Cc_* and *E. coli* BW25113 *ΔxylAB *:: Cm^R^ pCX-pL-*xylAXB_Cc_* pCL-*xylCD_Cc_* behaved identically with respect to substrate consumption and product formation (Table 2). These strains were only able to consume 39.8±0.03 % of the d-xylose, and accumulation of d-xylonolactone/d-xylonate was not detected. This confirms that *C. crescentus* XL and XD catalyze the efficient conversion of d-xylose to d-KDX both *in vitro* and *in vivo*. However, pyruvate accumulated to higher concentrations than any other strains, together with modest concentrations of acetate.

## Discussion

The utilization of pentose sugars *via* carbon-efficient metabolic routes is crucial to underpin future atom economy in the biomanufacturing of chemicals [1]. The Weimberg pathway [9] provides an efficient alternative to the classical PPP to convert d-xylose to TCA cycle intermediates, since it minimizes wasteful loss of carbon in the form of CO_2_ (Fig. 1). In this study, we successfully engineered an *E. coli* strain to use the Weimberg pathway constitutively by expressing the genes from *C. crescentus*, *xylAXB_Cc_*, from the constitutive pL promoter and deleting the native *xylAB_Ec_* genes. As a result, this strain was able to grow efficiently on d-xylose without the addition of glucose or other nutritional supplements. This opens the way to low-cost, atom-efficient, simplified bioprocesses to manufacture chemicals from waste biomass without any reliance on food-grade sugars or the need for multiple substrates and/or multiple feeding strategies during the fermentation. Constitutive expression also paves the way for future continuous biomanufacturing processes.

*E. coli* BW25113 *ΔxylAB *:: Cm^R^ pCX-pL-*xylAXB_Cc_* pCL was able to grow efficiently on d-xylose with an improved biomass yield compared with the wild-type host strain growing on d-xylose using the classical PPP, although the growth rate was lower. The increased growth yield is surprising because the PPP operating in the wild-type strain generates 31.7 mole ATP/mole d-xylose, whereas oxidation of d-xylose *via* the Weimberg pathway, TCA cycle, malic enzyme and pyruvate dehydrogenase generates only 30 mole ATP/mole d-xylose (Fig. S4). Therefore, further work is needed to explain the improved growth efficiency.

We obtained some evidence that the growth rate was compromised because of the maintenance of the recombinant plasmid, but multiple additional factors may also be involved. For example, it is possible that the metabolic burden of expressing multiple heterologous proteins could have an effect. Moreover, when we expressed *xylAXB_Cc_* using an IPTG-inducible system, a portion of the enzymes was insoluble and, for DPDH, we even had to decrease the temperature after induction to 18 °C for better protein yields (Fig. S5), a strategy that is widely used to improve the solubility of heterologous proteins [37]. This suggests that the folding of the enzymes from the Weimberg pathway is not optimal in *E. coli*. It is also possible that the growth rate was inhibited by transient accumulation of d-xylonolactone/d-xylonate, which is known to be toxic [31, 32].

We have also considered the possibility that an increased energy requirement for biosynthesis may have compromised the growth rate. Although a significant proportion of the biosynthetic requirements can be met through the utilization of 2-oxoglutarate and other TCA cycle intermediates for amino acid biosynthesis, the cells also need to produce biosynthetic intermediates from pyruvate, phosphoenol pyruvate and sugars (e.g. erythrose 4-phosphate and ribose 5-phosphate). In strains using the Weimberg pathway, these intermediates may need to be produced *via* gluconeogenesis, an ATP-dependent pathway, and it is possible that this may account for the decreased growth rate. However, this hypothesis does not explain the somewhat dramatic increase in the biomass yield during utilization of the Weimberg pathway compared with that for the wild-type strain, which can derive the same biosynthetic intermediates directly from the PPP and glycolysis, without any need for gluconeogenesis. Therefore, an alternative explanation is needed for these unusual and somewhat contradictory observations.

*E. coli* can metabolize d-KDX using aldolases encoded by *yjhH* and *yagE* (Fig. 1). These enzymes form the Dahms pathway, which converts d-KDX to pyruvate and glycolaldehyde [29, 38]. Pyruvate can enter the central metabolism either towards the generation of biosynthetic intermediates or *via* the pyruvate dehydrogenase reaction (thus producing acetyl-CoA). The glycolaldehyde is first converted to glycolate by the aldehyde dehydrogenase, aldA [39–41], which can be metabolized *via* either the glyoxylate shunt or the glyoxylate degradation pathway, which converts glyoxylate to tartronate semialdehyde, d-glycerate and then 2-phosphoglycerate [42]. Therefore, the Dahms pathway would provide intermediates for sugar biosynthesis without the requirement to produce phosphoenolpyruvate from TCA cycle intermediates. This, together with the direct supply of 2-oxoglutarate for energy generation *via* the Weimberg pathway, may explain the improved biomass yield compared with that for the wild-type cells relying solely on the PPP. Therefore, further work will be undertaken to confirm this hypothesis by measuring the carbon flux between the Weimberg and Dahms pathways.

In conclusion, to the best of our knowledge, we have developed the first *E. coli* strain able to grow constitutively on d-xylose *via* the Weimberg pathway as the sole carbon and energy source. The new strain has considerable potential for use as a host to produce fuels and chemicals from lignocellulosic sugars through the addition of new pathway modules to convert TCA cyle intermediates to industrially relevant targets, including amino acids [43], itaconate [44], succinate [45] or other di/tricarboxylic acids [21].

## Supplementary Data

1267 Supplementary File 1
