# Supplementary material for: Engineering Escherichia coli to grow constitutively on D-xylose using the carbon-efficient Weimberg pathway
Source: Microbiology (Reading). 2018 Feb 5;164(3):287–98. doi: 10.1099/mic.0.000611 (PMC5882109; doi:10.1099/mic.0.000611)
Supplement: Supplementary File 1 [file mic-164-287-s001.pdf]

Supplementary Material

Fig. S1: Preparation of phosphonate silyl ester (Ethyl 2-((tert-butyldimethylsilyl)oxy)-2-(dimethoxyphosphoryl)acetate) for the synthesis of 2-keto-3-deoxy-*D*-xylonate (*D*-KDX)

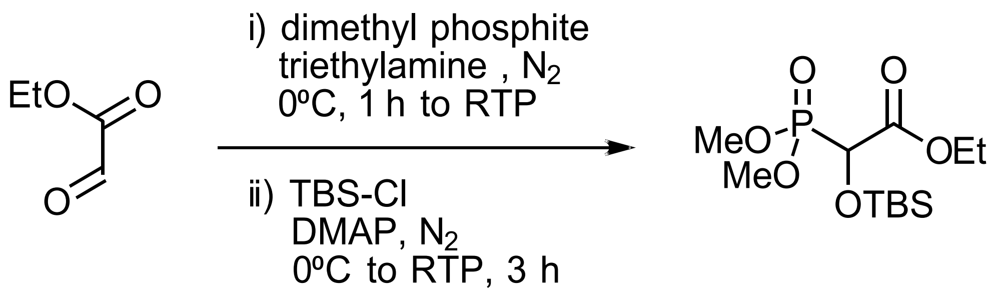

Dimethyl phosphite (1 mmol) was charged to a dried round-bottomed flask containing toluene (10 mL) at 0 °C under a blanket of nitrogen. Triethylamine (3 mmol) and then ethyl glyoxalate solution (50% in toluene, 1 mmol) were slowly charged to the agitated solution before allowing the flask to warm for 1 h to room temperature. Following formation of the alcohol (Rf = 0.10, 1:3 methanol:dichloromethane + 1% acetic acid) the reaction was cooled to 0 °C. Catalytic 4-dimethylaminopyridine (0.1 mmol) was then charged to the reaction vessel before dropwise addition of a solution of *tert*-butyldimethylsilyl chloride (2 mmol) in toluene (15 mL). The solution was allowed to warm to room temperature and stirred overnight before filtering off the salts and then washing with saturated ammonium chloride solution (50 mL), H_2O (2 x 25 mL) and brine (25 mL). The organic layer was then dried with Na_2SO_4, filtered, and the solvent removed by evaporation *in vacuo* to give a dark orange oil. The crude oil was then purified by silica column chromatography (2:1 ethyl acetate:hexane, Rf = 0.35) to give the title product as a pale yellow oil (70%). NMR analyses of the product are shown below.

^{1}H NMR (500 MHz, $CDCl_3$) δ_H = 4.53 (1H, d, J = 18.0, C*H*O), 4.25-4.14 (2H, m, OCH_2), 3.77 (3H, d, J = 4.9, OCH_A3), 3.74 (3H, d, J = 8.5, OCH_B3), 1.22 (3H, t, J = 7.2, CH2C*H*3), 0.84 (9H, s, C(C*H*3)3), 0.037 (3H, d, Si(CH_A3)), 0.027 (3H, d, Si(CH_B3));

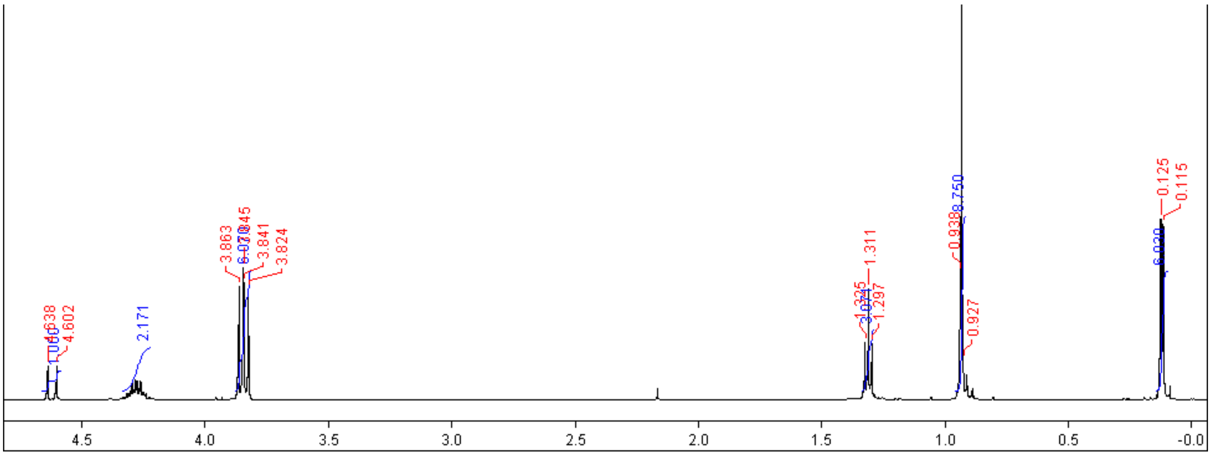

^{13}C NMR (125 MHz, $CDCl_3$) δ_C = 168 (d, J = 2.5), 70.6 (d, J = 162), 61.8, 54.0 (d, J = 7.0), 54.0 (d, J = 7.0), 25.5, 18.3, 14.0, -5.5 (d, J = 20.5);

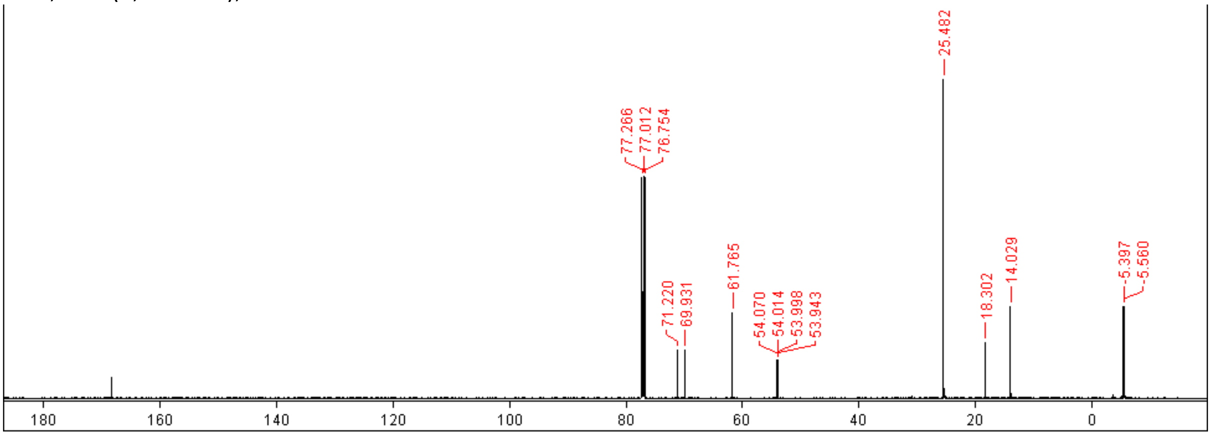

^{31}P (122 MHz, $CDCl_3$) δ_p = 18.0

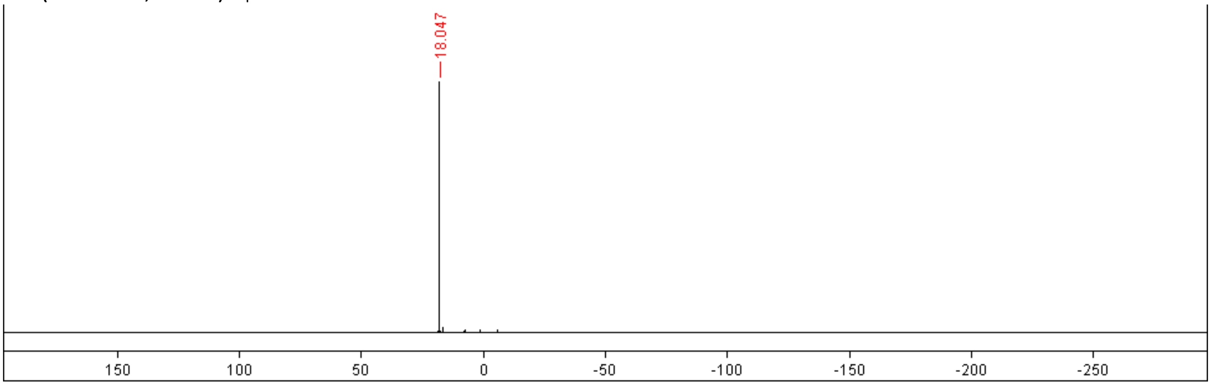

Fig. S2: Codon optimized gene sequences of *C. crescentus xylX*, *xylA*, *xylB*, *xylC* and *xylD* (*xylXABCD*$_{Cc}$)

Gene sequences for *xylX*$_{Cc}$ (a), *xylA*$_{Cc}$ (b), *xylB*$_{Cc}$ (c), *xylC*$_{Cc}$ (d) and *xylD*$_{Cc}$ (e) were codon optimized for expression in *E. coli* by Biomatik.

a.

```
1       ATGGGCGTGA GCGAATTTCT GCCAGAAGAT TGGAAAGCTG CAACATTATT AGGTCGTATC
61      GATTTTGGCG AAGGTCCTAC ACCTGTGCTG GTTCGCGGTG GTCGTGTTGA AGATGTGTCT
121     AAAATTGCAC CGACGGTTGC GGACTTGATG AATGCCTTTC AGCCGGGCGC AGTTATTCCT
181     CGCGGCGAAG ATAAAGGTCC ATTAGAAGCT CTGGATATAC GCCCGGTTTG GGAAGATCCG
241     GATGGTGCCG CTCCGGTTAA ACTGTTAGCT CCAGTGGATC TCCAGTGTCT GAAAGCCGCC
301     GGTGTGACGT TTGCCGTTTC AACGTTAGAG CGAGTTATCG AAGAACGCGC CCGTGGTGAC
361     GCAGGCGAAG CCCTGAAAAT TCGTACATTA TTAGCAGAAC GCATGGGTGG CGATCTGAAA
421     TCAGTGGAAC CGGGTAGTCA GGGCGCACAG CGCTTAAAAG ATGCCTTAAT CGCCGATGGC
481     TTATGGAGTC AGTATCTGGA AGTTGCTATC GGCCCGGATG CCGAAATCTT TACCAAAGGT
541     CCTACCCTGT CTAGTATGGG CTGGGGGGAT CAGGTTGGCG TTCGCTATGA TAGTCATTGG
601     AACAACCCAG AACCAGAAGT TGTTCTGCTG TGCGATGGTA GCGGCTTAAT CCGTGGTGCC
661     GCATTAGGCA ATGATGTTAA CTTGCGCGAT TTTGAAGGCC GCTCAGCCTT ATTACTGAGC
721     AAAGCCAAAG ATAATAACGC ATCTTGTGCT ATCGGTCCAT TCTTTCGCCT GTTTGATGAA
781     ACCTTTGGCT TAGATGATGT GCGCTCTGCG GAAGTTGAAC TGAAAATCAC GGGTCGTGAT
841     AATTTTGTGT TAGATGGCAA ATCTAATATG TCACTGATCT CACGCGATCC AGCAGTTCTG
901     GCAGGCCAGG CATACGGTAA ACAGCATCAG TATCCGGATG GCTTTGCACT GTTTCTGGGG
961     ACGATGTTTG CACCTATTCA GGATCGTGAT ACACCGGGCC AGGGCTTTAC ACATAAAGTG
1021    GGCGATCGCG TTCGTGTGAG TACCCCTAAA CTGGGTGTGC TGGAAAACGA AGTGACAACA
1081    TGCGATAAAG CCAAACCTTG GACGTTTGGC ATCTCAGCAC TGATTCGTAA CCTAGCGGGT
1141    CGCGGCCTGC TGTAA
```

b.

```
1       ATGACGGATA CATTACGCCA TTATATCGGC GGCGAGAGAG TGGCTGCCGA TGCTCCTGCG
61      GAATCTCTGA ATCCAAGCAA TACCAATGAT GTGGTTGCTA AAGTTCCTAT GGGCGGTCAG
121     GCAGAAGTTG ATGCCGCAGT TGATGCCGCG AGGAAAGCCT TTCCTGCTTG GGCCGATGCT
181     TCTCCGGAAG TTCGTAGCGA TCTGTTAGAT AAAGTGGGTA GTACCATCAT TGCACGCTCA
241     GCCGATATTG GTCGTCTGTT AGCACGCGAA GAAGGAAAGA CCTTAGCGGA AGGCATCGGT
301     GAAACGGTTC GCGCAGGTCG TATCTTTAAA TATTTTGCAG GTGAAGCATT ACGTCGTCAT
361     GGTCAGAATC TCGAATCTAC ACGTCCGGGT GTTGAAATTC AGACATATCG TCAGGCAGTG
421     GGTGTGTATG GTTTAATCAC CCCTTGGAAT TTTCCTATTG CCATCCCGGC TTGGAAAGCA
481     GCTCCAGCCT TAGCCTTTGG CAATACCGTT GTGATCAAAC CTGCGGGTCC TACACCTGCG
541     ACCGCCAATG TTCTGGCAGA TATAATGGCC GAATGCGGCG CTCCAGCAGG CGTGTTTAAT
601     ATGTTATTTG GTCGCGGTAG TATGGGCGAT GCCCTGATCA AACATAAAGA TGTGGATGGC
661     GTGAGCTTTA CGGGCTCACA GGGTGTGGGT GCACAGGTTG CTGCGGCGGC TGTTGCCCGC
721     CAGGCTCGTG TTCAGTTAGA AATGGGTGGC AAAAATCCAC TGATTGTGCT GGATGATGCG
781     GACCTAGAAC GTGCTGTGGC TATTGCATTA GATGGTAGCT TTTTCGCCAC CGGCCAGCGC
841     TGTACCGCCT CTAGTCGTCT GATTGTTCAG GATGGTATTC ATGATAAATT TGTCGCATTA
901     CTCGCTGAAA AAGTGGCCGC ATTGCGGGTG GGCGATGCAT TAGATCCTAA TACACAGATC
961     GGCCCAGCTG TGAGCGAAGA TCAGATGGAA ACGAGTTATC GCTATATCGA CATCGCTGCC
1021    TCTGAAGGTG GTCGCGTTGT GACGGGCGGC GATCGCATCA AACTGGATAA TCCGGGCTGG
1081    TATGTTCGTC CGACACTGAT TGCGGATACA CAGGCCGGAA TGCGCATCAA TAACGAGGAA
1141    GTGTTTGGTC CAGTGGCTTC TACCATTCGC GTTAAATCTT ATGAAGAAGC CCTGGAAATT
1201    GCTAATGGCG TTGAATTTGG TCTGAGCGCG GGCATCGCCA CGACGAGTCT GAAACATGCC
1261    CGTCATTTTC AGCGCTATGC GCGCGCGGGT ATGACAATGG TTAACTTGGC AACCGCCGGC
1321    GTTGATTATC ATGTTCCGTT TGGCGGCACG AAATCTAGTA GCTATGGTGC TCGCGAACAG
1381    GGCTTTGCAG CCGTTGAATT TTTCACCCAG ACCAAAACCT CTTATTCTTG GAGTTAA
```

c.

```
1       ATGTCAAGTG CCATCTATCC GAGCTTAAAA GGCAAGAGAG TGGTTATTAC GGGCGGTGGC
61      TCAGGCATCG GCGCAGGTCT GACAGCGGGC TTTGCCCGTC AGGGCGCCGA AGTTATCTTT
121     CTGGATATTG CTGATGAAGA TAGTCGCGCA CTGGAAGCGG AACTGGCCGG CTCTCCAATC
181     CCTCCAGTGT ACAAACGCTG CGATCTCATG AATCTGGAAG CAATCAAAGC AGTGTTTGCA
241     GAAATCGGCG ACGTGGATGT GTTAGTTAAC AACGCAGGTA ATGATGATCG TCATAAATTA
301     GCCGATGTGA CCGGTGCCTA TTGGGATGAA CGTATCAATG TGAACCTGCG TCACATGCTG
361     TTTTGTACAC AGGCCGTTGC TCCGGGTATG AAAAAACGCG GCGGTGGTGC AGTTATCAAT
421     TTTGGTAGTA TCTCTTGGCA TCTGGGTTTA GAGGATCTAG TGCTGTATGA AACCGCTAAA
481     GCCGGCATCG AAGGCATGAC TCGCGCCTTA GCCCGCGAAC TGGGTCCAGA TGATATTCGC
541     GTGACCTGCG TTGTTCCTGG TAATGTTAAA ACCAAACGCC AGGAAAAATG GTATACACCG
601     GAAGGCGAAG CTCAGATCGT TGCAGCACAG TGTCTGAAAG GTCGCATTGT TCCAGAAAAT
661     GTGGCCGCCT TAGTTCTGTT TCTGGCATCT GATGATGCGA GCTTATGCAC GGGCCATGAA
721     TATTGGATCG ATGCGGGTTG GCGTTAA
```

d.

```
1      ATGACAGCAC AGGTGACGTG CGTGTGGGAT TTGAAAGCCA CCTTAGGTGA AGGCCCAATC
61     TGGCATGGCG ATACACTGTG GTTTGTTGAT ATTAAACAGC GCAAAATTCA TAATTATCAT
121    CCTGCTACGG GCGAACGCTT TTCTTTTGAT GCCCCGGATC AGGTGACATT TCTGGCACCA
181    ATCGTTGGCG CGACGGGTTT TGTTGTGGGC CTGAAAACGG GTATTCATCG CTTTCATCCG
241    GCAACGGGCT TTTCTCTGCT GCTGGAAGTG GAAGATGCCG CGCTGAATAA CCGTCCTAAT
301    GATGCCACAG TGGATGCGCA GGGTCGCCTG TGGTTTGGTA CAATGCATGA TGGTGAAGAA
361    AACAACAGCG GCTCACTGTA TCGTATGGAT CTGACCGGTG TTGCTCGTAT GGATCGCGAC
421    ATCTGCATCA CGAATGGCCC TTGCGTGTCT CCAGATGGCA AAACATTTTA TCATACCGAT
481    ACGTTAGAAA AAACAATCTA TGCGTTTGAC TTGGCCGAAG ATGGCTTACT GAGTAATAAG
541    CGCGTGTTTG TGCAGTTTGC ACTGGGCGAT GATGTGTATC CGGATGGTAG CGTTGTGGAT
601    TCAGAAGGCT ATCTTTGGAC CGCCTTATGG GGCGGCTTTG GTGCGGTTCG CTTTAGTCCT
661    CAGGGAGACG CCGTTACACG CATCGAATTA CCAGCTCCTA ATGTTACCAA ACCTTGCTTT
721    GGCGGTCCGG ACCTGAAAAC CTTATATTTT ACGACCGCAA GGAAAGGCCT GTCAGATGAA
781    ACATTAGCTC AGTATCCGTT AGCAGGCGGC GTGTTTGCAG TTCCAGTTGA TGTTGCCGGT
841    CAGCCACAGC ATGAAGTTCG TCTGGTTTAA
```

e.

```
1      ATGTCAAATC GTACACCACG TCGCTTTCGC TCACGCGATT GGTTTGATAA TCCGGATCAT
61     ATCGATATGA CAGCACTGTA TTTGGAACGT TTTATGAATT ATGGCATCAC CCCAGAAGAA
121    TTACGCTCAG GCAAACCTAT TATCGGCATT GCCCAGACGG GCTCAGACAT ATCTCCTTGT
181    AATCGCATTC ATCTGGACCT GGTTCAGCGT GTTCGTGATG GCATCCGTGA TGCAGGTGGT
241    ATTCCTATGG AATTTCCAGT TCATCCGATC TTTGAAAATT GTCGTCGTCC TACTGCGGCC
301    TTAGATCGTA ATCTGAGCTA TCTGGGCTTA GTGGAAACCT TACATGGCTA TCCAATCGAT
361    GCAGTTGTGC TGACGACCGG TTGCGATAAA ACAACACCAG CAGGCATTAT GGCTGCTACG
421    ACCGTTAATA TCCCGGCCAT TGTTCTGAGC GGCGGTCCTA TGTTAGATGG TTGGCATGAA
481    AACGAACTGG TGGGTAGCGG GACAGTTATT TGGCGCTCTC GTCGCAAATT AGCAGCCGGC
541    GAAATCACGG AGGAGGAGTT CATTGATCGC GCCGCTTCTA GTGCCCCGAG CGCCGGTCAT
601    TGTAATACGA TGGGTACGGC CTCTACCATG AATGCAGTTG CGGAAGCACT GGGCCTGAGT
661    CTGACGGGTT GCGCCGCTAT TCCTGCTCCT TATCGCGAAC GTGGTCAGAT GGCCTACAAG
721    ACGGGTCAGC GCATTGTGGA TCTGGCCTAT GATGATGTTA AACCTCTGGA TATTCTGACC
781    AAACAGGCTT TTGAAAATGC AATTGCGTTA GTTGCTGCTG CGGGCGGTTC AACCAATGCA
841    CAGCCACATA TTGTTGCAAT GGCCCGCCAT GCTGGTGTGG AAATCACCGC GGATGATTGG
901    CGCGCAGCGT ATGATATTCC ACTGATTGTG AATATGCAGC CTGCGGGCAA ATACCTAGGC
961    GAACGTTTTC ATCGTGCCGG CGGTGCTCCA GCTGTGCTGT GGGAACTGTT ACAGCAGGGT
1021   CGCTTACATG GTGACGTTCT GACAGTGACC GGGAAAACCA TGTCAGAAAA TCTTCAGGGT
1081   CGCGAAACAA GCGATCGCGA AGTGATCTTT CCGTATCATG AACCTTTAGC CGAAAAAGCC
1141   GGCTTTTTAG TGTTAAAAGG TAATCTGTTT GATTTTGCCA TCATGAAAAG TAGCGTGATC
1201   GGCGAAGAAT TTCGTAAACG CTATCTGTCT CAGCCAGGTC AGGAAGGTGT GTTTGAAGCA
1261   CGCGCCATTG TGTTTGATGG TAGCGATGAT TATCATAAAC GTATTAATGA TCCAGCCCTG
1321   GAAATCGATG AACGCTGTAT CTTAGTTATT CGCGGTGCGG GCCCAATCGG TTGGCCAGGC
1381   TCTGCCGAAG TGGTTAATAT GCAGCCTCCG GATCATCTGC TGAAAAAAGG CATTATGTCT
1441   TTACCGACAC TGGGCGACGG TCGTCAGTCA GGTACAGCCG ATAGTCCGAG TATTCTGAAT
1501   GCAAGTCCAG AAAGTGCTAT CGGTGGCGGC CTGAGTTGGT TACGCACGGG CGATACCATC
1561   CGTATCGATC TCAATACAGG TCGTTGCGAT GCCCTGGTGG ATGAAGCTAC GATTGCCGCA
1621   CGCAAACAGG ATGGCATCCC TGCCGTTCCT GCAACAATGA CCCCTTGGCA GGAAATCTAT
1681   CGTGCACATG CGTCTCAGTT AGATACGGGC GGCGTTCTGG AATTTGCCGT TAAATATCAG
1741   GACCTGGCTG CCAAACTGCC TCGCCATAAT CATTAA
```

Fig. S3: Sequences of promoters used to generate the pCX libraries

The sequence of the promoters pL (a), pR (b), pAspC (c), and pOSMY (d) are shown below, together with the related references from which they were taken.

a.

```
  1  CGCGTAGGAT CGAGATCTAA GCTGTTGTGA CCGCTTGCTC TAGCCAGCTA TCGAGTTGTG
 61 AACCGATCCA TCTAGCAATT GGTCTCGATC TAGCGATAGG CTTCGATCTA GCTATGTAGA
121 AACGCCGTGT GCTCGATCGC TCTAACACCG TGCGTGTTGA CTATTTTACC TCTGGCGGTG
181 ATAATGGTTG CATGTACTAA GGAGGATCGC CATG
```

Reference:

Cheng X. and Patterson TA. 1992, Construction and use of lambda pL promoter vectors for direct cloning and high level expression of PCR amplified DNA coding sequences, *Nucleic Acids Research,* **20:** 4591-98.

b.

```
  1  AGGATCGAGA TCTAAGCTGT TGTGACCGCT TGCTCTAGCC AGCTATCGAG TTGTGAACCG
 61 ATCCATCTAG CAATTGGTCT CGATCTAGCG ATAGGCTTCG ATCTAGCTAT GTAGAAACGC
121 CGTGTGCTCG ATCGCTTGAT AAGGTCCACG TAGCTGCTAT AATTGCTTCA ACAGAACATA
181 TTGACTATCC GGTATTACCC GGCAGATCGT TGTCGATCCT ACCATCCACT CGACACACCC
241 GCCAGCGGCC GCTGCCAAGC TTCCGAGCTC TCGAATTCAA AGGAGGTACC CGCCATG
```

Reference:

Weisemann JM and Weinstock GM. 1991, The promoter of the *recA* gene of *Escherichia coli*, *Biochimie,* **73:** 457-70.

c.

```
  1  GACGTTCTGT TAGTTGCGCA ATACCAGTTC GATTTCGGTC TGCGTCCGTC CATCGCTTAC
 61 ACCAAATCTA AAGCGAAAGA CGTAGAAGGT ATCGGTGATG TTGATCTGGT GAACTACTTT
121 GAAGTGGGCG CAACCTACTA CTTCAACAAA AACATGTCCA CCTATGTTGA CTACATCATC
181 AACCAGATCG ATTCTGACAA CAAACTGGGC GTAGGTTCAG ACGACACCGT TGCTGTGGGT
241 ATCGTTTACC AGTTCTAATA GCACACCTCT TTGTTAAATG CCGAAAAAAC AGGACTTTGG
301 TCCTGTTTTT TTTATACCTT CCAGAGCAAT CTCACGTCTT GCAAAAACAG CCTGCGTTTT
361 CATCAGTAAT AGTTGGAATT TTGTAAATCT CCCGTTACCC TGATAGCGGA CTTCCCTTCT
421 GTAACCATAA TGGAACCTCG CCATG
```

Reference:

Fotheringham I, Taylor PP and Yoshida RK. 1996, Materials and methods for the production of *D*-phenylalanine, Patent EP736604A2.

d.

```
  1  CACCAGGCGT TTAAGGGCAC CAACGCGTCT GGCACAGGAA CGTTATCCGG ACGTTCAGTT
 61 CCACCAGACC CGCGAGCATT AATTCTTGCC TCCAGGGCGC GGTAGTGGCG CCCTGTCAAT
121 TTCCCTTCCT TATTAGCCGC TTACGGAATG TTCTTAAAAC ATTCACTTTT GCTTATGTTT
181 TCGCTGATAT CCCGAGCGGT TTCAAAATTG TGATCTATAT TTAACAAAGG AGGAGGATCG
241 CCATG
```

Reference:

Yim HH, Brems RL and Villarejo M. 1994, Molecular characterization of the promoter of osmY, an rpoS-dependent gene, *J Bacteriol,* **176:** 100-07.

Fig. S4: Stoichiometry for oxidation of *D*-xylose

Stoichiometry for *D*-xylose oxidation *via* the pentose phosphate (a) and the Weimberg pathway (b).

a.

Oxidation:

Pentose phosphate pathway

3 Xylose + 5 ATP →
5 Glyceraldehyde 3P + 5 ADP

Glycolysis

5 Glyceraldehyde 3P + 10 ADP + 5 P + 10 NAD + 5 CoASH →
5 Acetyl CoA + 5 CO_2 + 10 ATP + 10 NADH + 5H_2O

TCA cycle

5 Acetyl CoA + 5 ADP + 5 P + 5 NADP + 10 NAD + 5 UQ + 10 H_2O→
10CO_2 + 5 CoASH + 5 ATP + 5 NADPH + 10 NADH + 10H^+ + 5 UQH_2.

Cancelling:

3 Xylose + 5 ATP →
~~5 Glyceraldehyde 3P~~ + 5 ADP

~~5 Glyceraldehyde 3P~~ + 10 ADP + 5 P + 10 NAD + ~~5 CoASH~~ →
~~5 Acetyl CoA~~ + 5 CO_2 + 10 ATP + 10 NADH + 5H_2O

~~5 Acetyl CoA~~ + 5 ADP + 5 P + 5 NADP + 10 NAD + 5 UQ + 10 H_2O→
10CO_2 + ~~5 CoASH~~ + 5 ATP + 5 NADPH + 10 NADH + 10H^+ + 5 UQH_2.

Combining:

3 Xylose + 5 ATP + 10 ADP + 5 P + 10 NAD + 5 ADP + 5 P + 5 NADP + 10 NAD + 5 UQ + 10 H_2O → 5 ADP + 5 CO_2 + 10 ATP + 10 NADH + 5H_2O + 10CO_2 + 5 ATP + 5 NADPH + 10 NADH + 10H^+ + 5 UQH_2.

Simplifying:

3 Xylose + 10 ADP + 10 P + 20 NAD + 5 NADP + 5 UQ + 5 H_2O →
15 CO_2 + 10 ATP + 20 NADH + 5 NADPH + 10H^+ + 5 UQH_2

ATP yield:

25 NADH = 75 ATP
5 UQ = 10 ATP
10 ATP

Total ATP = 95 (31.66 ATP/xylose)

b.

<u>Oxidation:</u>

Weimberg pathway

Xylose + 2 NADP →
2-oxoglutarate + 2 NADPH + 3 H^+

Oxidation of 2-oxoglutarate via the TCA cycle and malic enzyme

2-oxoglutarate + 3 NAD + 2 CoASH + ADP + P + UQ + H_2O→
Acetyl CoA + 3 CO_2 + CoASH + 3 NADH + ATP + UQH_2

TCA cycle

Acetyl CoA + ADP + P + NADP + 2 NAD + UQ + 2 H_2O→
2 CO_2 + CoASH + ATP + NADPH + 2 NADH + 2 H^+ + UQH_2.

<u>Cancelling:</u>

Xylose + 2 NADP →
~~2-oxoglutarate~~ + 2 NADPH + 3 H^+
~~2-oxoglutarate~~ + 3 NAD + 2 CoASH + ADP + P + UQ + H_2O→
~~Acetyl CoA~~ + 3 CO_2 + CoASH + 3 NADH + ATP + UQH_2
~~Acetyl CoA~~ + ADP + P + NADP + 2 NAD + UQ + 2 H_2O→
2 CO_2 + CoASH + ATP + NADPH + 2 NADH + 2 H^+ + UQH_2

<u>Combining:</u>

Xylose + 2 NADP + 3 NAD + 2 CoASH + ADP + P + UQ + H_2O + ADP + P + NADP + 2 NAD + UQ + 2 H_2O →
2 NADPH + 3 H^+ + 3 CO_2 + CoASH + 3 NADH + ATP + UQH_2 + 2 CO_2 + CoASH + ATP + NADPH + 2 NADH + 2 H^+ + UQH_2

<u>Simplifying:</u>

Xylose + 3 NADP + 5 NAD + 2ADP + 2P + 2UQ + 3H_2O →
5CO_2 + 3 NADPH + 5 H^+ + 5 NADH + 2ATP + 2UQH_2

<u>ATP yield:</u>

8 NADH = 24 ATP
2UQ = 4 ATP
2ATP

Total ATP/xylose = 30

Fig. S5: SDS-PAGE analysis of expression of *C. crescentus xylX*, *xylA*, *xylB*, *xylC* and *xylD* (*xylXABCD$_{Cc}$*)

SDS-Polyacrylamide Gel Electrophoresis (SDS-PAGE) was used to analyze the soluble and insoluble fractions of the cell-free extracts of *E. coli* BL21(DE3) pLysS pET-20b(+)*xylX$_{Cc}$*, pET-20b(+)*xylA$_{Cc}$*, pET-20b(+)*xylB$_{Cc}$*, pET-20b(+)-*xylC$_{Cc}$*, pET-20b(+)-*xylD$_{Cc}$*. For expression of *xylB$_{Cc}$*, *xylX$_{Cc}$* and *xylC$_{Cc}$*, cells were grown at 37 °C and then, after induction, moved to 30 °C (b). For expression *xylD$_{Cc}$* and *xylA$_{Cc}$*, cells were moved from 37 to 18 °C (a). *Ladder* refers to a mixture of proteins having defined molecular weights (indicated in kDa) which allowed mass evaluation of unknown proteins by comparing migration. *Control* refers to a soluble protein fraction extracted from *E. coli* BL21(DE3)pLysS transformed with pET20b(+) not containing the genes of interest. For analysis of expression of each gene, both soluble and insoluble fractions at 0 h, 5 h and overnight (O/N) after induction with IPTG are shown. The level of migration corresponding to the enzymes molecular weight is circled in black.

a.

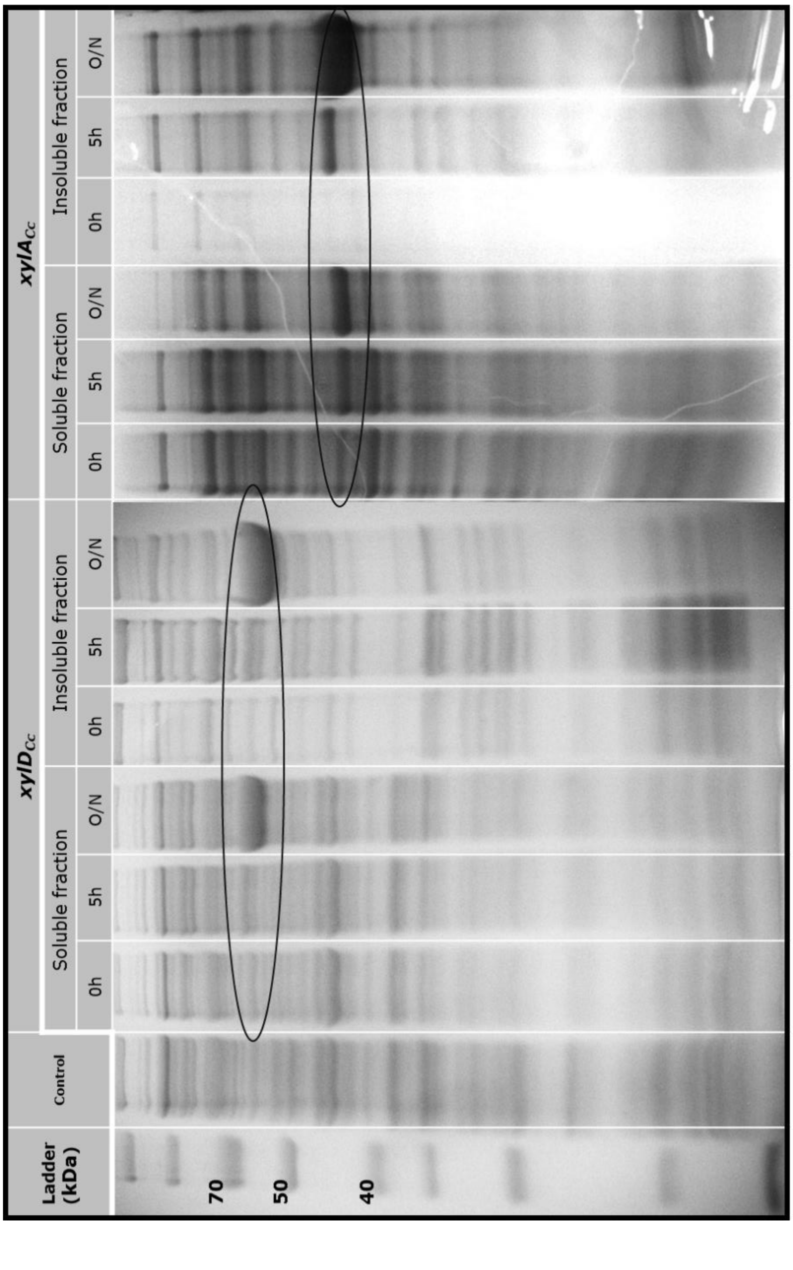

b.

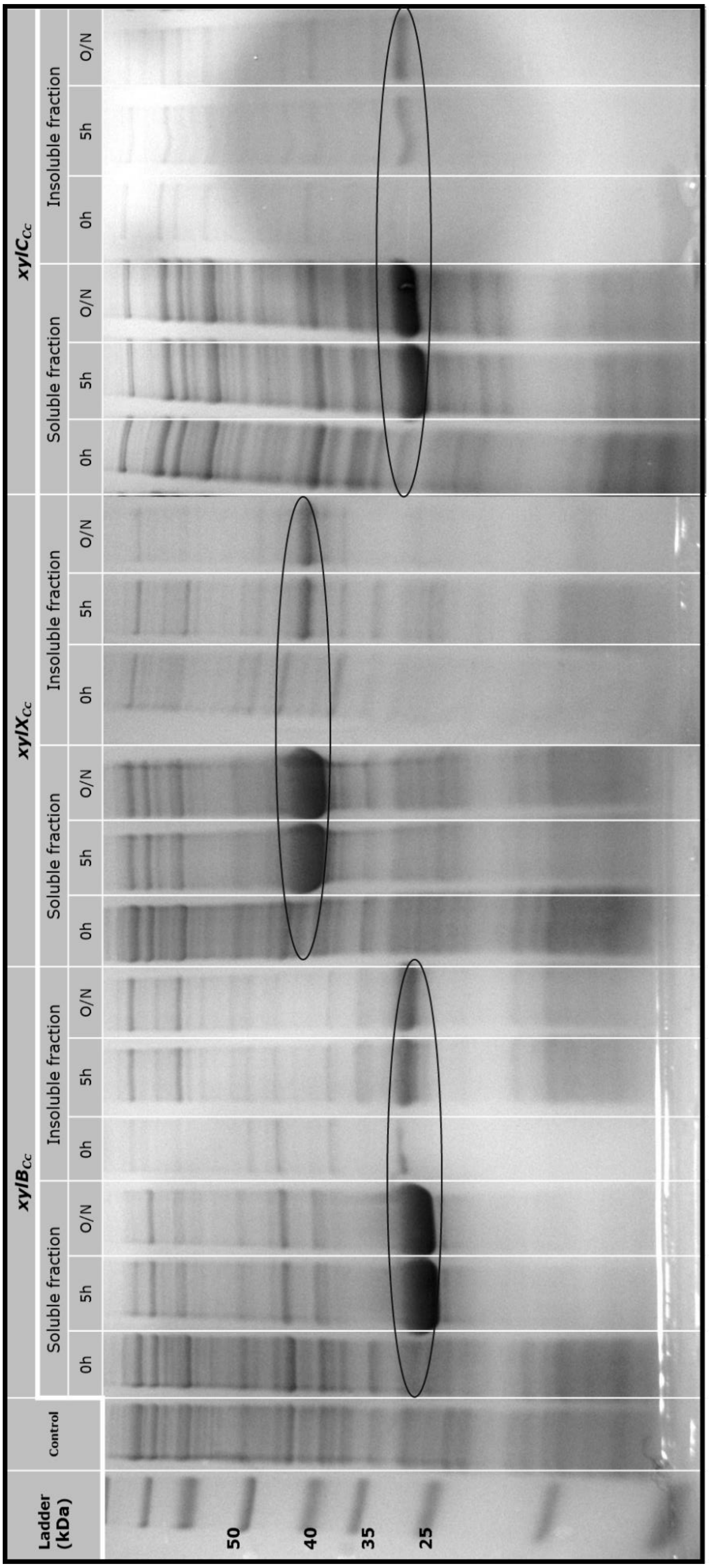
Ladder (kDa)
50
40
35
25
Control
xylBCc
xylXCc
xylCCc
Soluble fraction
Insoluble fraction
0h
5h
O/N

Fig. S6: Agar plate screening

(a) and (b) refer to *E. coli* BW25113 pCX and *E. coli* BW25113 *Δicd ΔxylAB::*Cm^{R} pCX, respectively. (c) to (h) refers to *E. coli* BW25113 *Δicd ΔxylAB::*Cm^{R} transformed with plasmid libraries 1 to 6, respectively. Cells were plated on ML medium supplemented with *D*-xylose (10 g/L) and Kanamycin (ML-XKm).

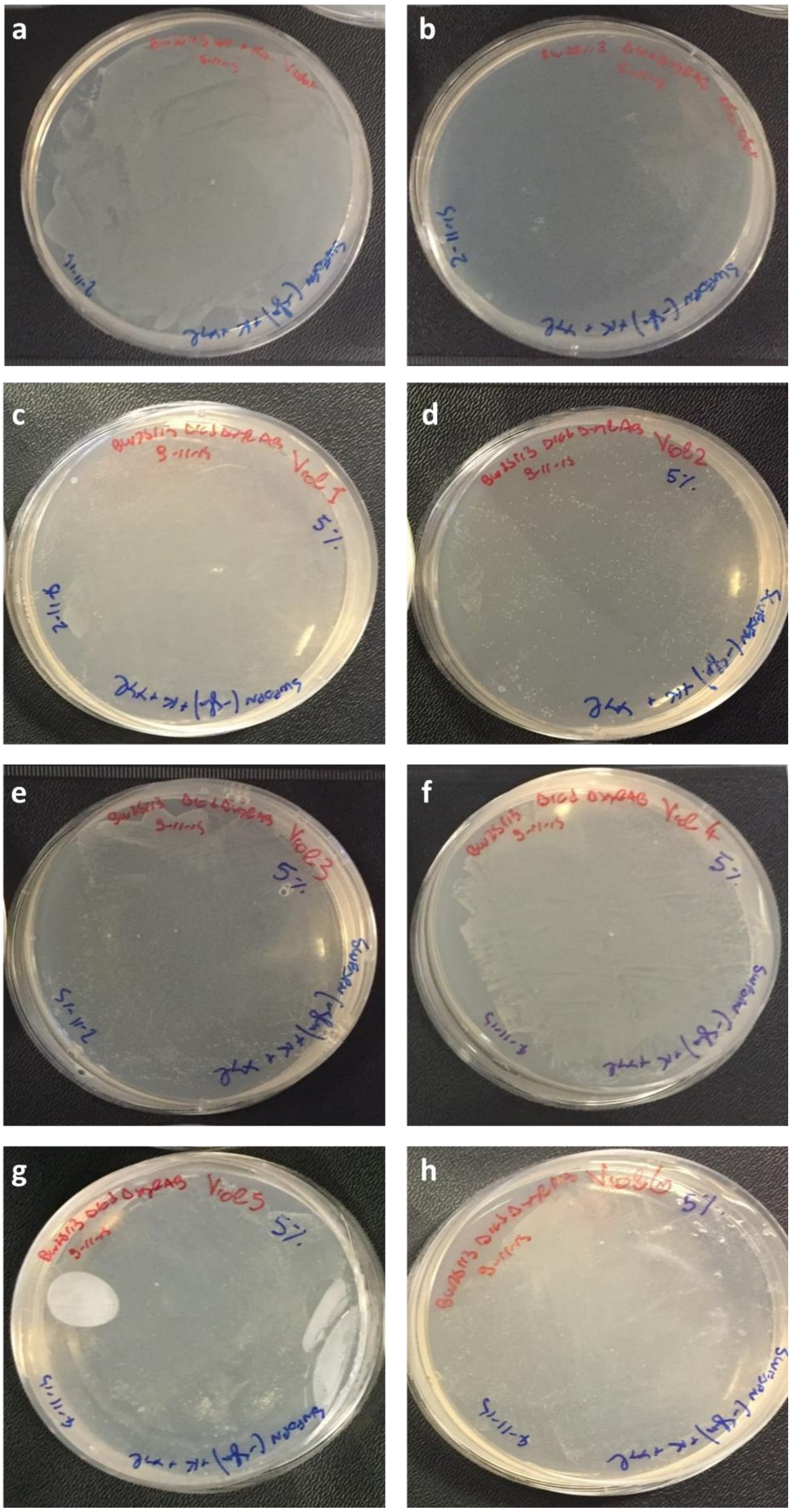

Fig S7: Growth in shake flask cultures of *E. coli* BW25113 *Δicd ΔxylAB*::Cm^R pCX-pL-xylABX$_{Cc}$ and *E. coli* BW25113 *Δicd ΔxylAB*::Cm^R pCX-pL-*xylAXB$_{Cc}$*

E. coli strains were grown in flask (100 mL culture volume) in ML medium supplemented with (a) *D*-xylose (10 g/L) or (b) *D*-glucose (10 g/L). A control was also analyzed, consisting of *E. coli* BW25113 pCX. Dry Cell Weight (DCW) and the growth rate for each strain (*μ*) is indicated. Assays were done in triplicate, and the means and standard errors are shown.

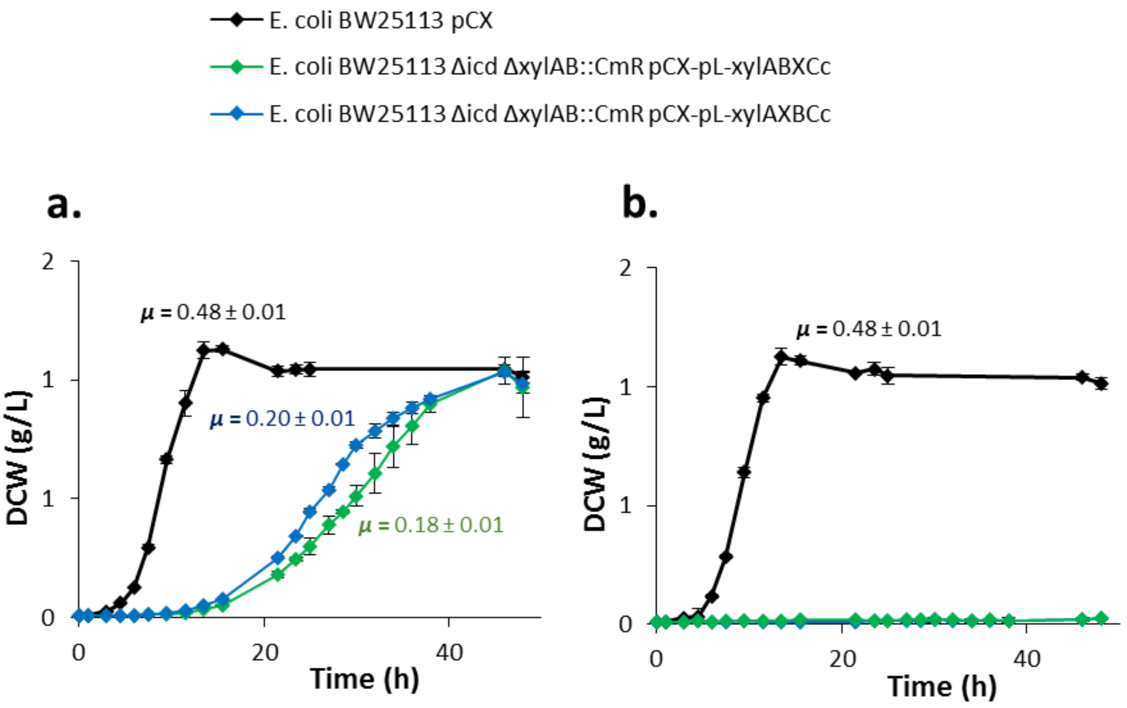

Table S8: Full metabolite data of strains described in Table 3 and Figure 5 in the text

Cells were grown in flask (100 mL culture volume) and presence of *D*-xylose, acetate, lactate, formate, succinate, ethanol, 2-oxoglutarate, pyruvate, *D*-KDX and *D*-xylonolactone/*D*-xylonate was analyzed at intervals. Assays were done in triplicate, and the means and standard errors are shown.

E. coli **BW25113 pCX pCL**

Fig. 5c in text

Time (h)	Ethanol g/L		Acetate g/L		Lactate g/L		Formate g/L		Succinate g/L		2-oxoglutarate g/L		*D*-Xylose g/L		Pyruvate g/L		*D*-Xylonate/ *D*-Xylonolactone g/L		*D*-KDX g/L	
	AVG	SD	AVG	SD	AVG	SD	AVG	SD	AVG	SD	AVG	SD	AVG	SD	AVG	SD	AVG	SD	AVG	SD
0	0.00	0.00	0.00	0.00	0.00	0.00	0.00	0.00	0.00	0.00	0.00	0.00	11.51	0.01	0.00	0.00	0.00	0.00	0.00	0.00
8	0.02	0.03	0.27	0.01	0.02	0.01	0.08	0.03	0.00	0.00	0.31	0.01	10.14	0.09	0.01	0.00	0.00	0.00	0.00	0.00
11	0.29	0.01	1.01	0.01	0.97	0.04	0.67	0.02	0.02	0.03	0.45	0.01	6.42	0.10	0.48	0.01	0.00	0.00	0.00	0.00
13	0.39	0.01	1.38	0.01	1.71	0.03	0.72	0.03	0.08	0.01	0.45	0.01	4.67	0.28	1.23	0.03	0.00	0.00	0.00	0.00
24	0.34	0.01	3.64	0.05	0.02	0.01	0.64	0.01	0.01	0.01	0.22	0.01	2.06	0.03	0.71	0.01	0.00	0.00	0.00	0.00
48	0.31	0.01	4.76	0.04	0.01	0.01	0.64	0.01	0.01	0.01	0.02	0.01	0.87	0.02	0.02	0.01	0.00	0.00	0.00	0.00
59	0.29	0.01	4.80	0.04	0.01	0.01	0.65	0.01	0.01	0.01	0.01	0.01	0.86	0.01	0.01	0.01	0.00	0.00	0.00	0.00

E. coli **BW25113 *Δicd ΔxylAB::*Cm**[R] **pCX-pL-*xylAXB*$_{Cc}$ pCL**

Fig. 5a in text

Time (h)	Ethanol g/L		Acetate g/L		Lactate g/L		Formate g/L		Succinate g/L		2-oxoglutarate g/L		*D*-Xylose g/L		Pyruvate g/L		*D*-Xylonate/ *D*-Xylonolactone g/L		*D*-KDX g/L	
	AVG	SD	AVG	SD	AVG	SD	AVG	SD	AVG	SD	AVG	SD	AVG	SD	AVG	SD	AVG	SD	AVG	SD
0	0.00	0.00	0.00	0.00	0.00	0.00	0.00	0.00	0.00	0.00	0.00	0.00	11.85	0.03	0.00	0.00	0.00	0.00	0.00	0.00
8	0.00	0.00	0.00	0.00	0.00	0.00	0.00	0.00	0.00	0.00	0.01	0.01	12.00	0.01	0.00	0.00	0.00	0.00	0.00	0.00
11	0.00	0.00	0.00	0.00	0.00	0.00	0.00	0.00	0.00	0.00	0.01	0.01	11.97	0.01	0.00	0.00	0.00	0.00	0.00	0.00
31	0.00	0.00	0.44	0.02	0.00	0.00	0.01	0.01	0.30	0.00	0.11	0.01	9.33	0.04	0.00	0.00	0.00	0.00	0.00	0.00
41	0.00	0.00	1.36	0.10	0.00	0.00	0.05	0.01	0.98	0.00	0.04	0.01	5.34	0.18	0.00	0.00	1.35	0.14	0.00	0.00
55.75	0.00	0.00	2.23	0.05	0.00	0.00	0.08	0.01	0.79	0.00	0.06	0.01	0.00	0.00	0.14	0.01	2.73	0.01	0.00	0.00
59	0.00	0.00	2.25	0.01	0.00	0.00	0.07	0.01	0.61	0.00	0.06	0.01	0.00	0.00	0.14	0.01	2.85	0.16	0.00	0.00

E. coli **BW25113 *ΔxylAB::*Cm**[R] **pCX-pL-*xylAXB*$_{Cc}$ pCL**

Fig. 5d in text

Time (h)	Ethanol g/L	Acetate g/L	Lactate g/L	Formate g/L	Succinate g/L	2-oxoglutarate g/L	*D*-Xylose g/L	Pyruvate g/L	*D*-Xylonate/ *D*-Xylonolactone g/L	*D*-KDX g/L

	AVG	SD	AVG	SD	AVG	SD	AVG	SD	AVG	SD	AVG	SD	AVG	SD	AVG	SD	AVG	SD	AVG	SD
0	0.00	0.00	0.00	0.00	0.00	0.00	0.00	0.00	0.00	0.00	0.00	0.00	10.82	0.00	0.00	0.00	0.00	0.00	0.00	0.00
8	0.00	0.00	0.00	0.00	0.00	0.00	0.00	0.00	0.00	0.00	0.00	0.00	10.77	0.08	0.00	0.00	0.00	0.00	0.00	0.00
16	0.00	0.00	0.00	0.00	0.00	0.00	0.00	0.00	0.00	0.00	0.00	0.00	10.75	0.06	0.00	0.00	0.00	0.00	0.00	0.00
26	0.00	0.00	0.25	0.17	0.00	0.00	0.00	0.00	0.15	0.07	0.00	0.00	8.88	0.51	0.00	0.00	1.69	0.01	0.00	0.00
29	0.00	0.00	1.05	0.41	0.05	0.07	0.12	0.04	0.36	0.14	0.00	0.00	6.61	0.79	0.00	0.00	2.43	0.71	0.00	0.00
32	0.00	0.00	1.93	0.36	0.17	0.08	0.24	0.04	0.55	0.11	0.00	0.00	4.25	0.58	0.00	0.00	2.29	0.61	0.00	0.00
35	0.00	0.00	2.92	0.48	0.20	0.01	0.28	0.04	0.66	0.10	0.00	0.00	1.72	0.54	0.00	0.00	2.10	0.75	0.00	0.00
38	0.00	0.00	3.49	0.31	0.14	0.04	0.28	0.01	0.71	0.01	0.00	0.00	0.53	0.74	0.00	0.00	1.15	0.01	0.00	0.00
50	0.00	0.00	3.15	0.50	0.00	0.00	0.29	0.01	0.25	0.27	0.00	0.00	0.00	0.00	0.00	0.00	0.29	0.04	0.00	0.00
60.25	0.00	0.00	2.75	0.03	0.00	0.00	0.30	0.01	0.04	0.01	0.00	0.00	0.00	0.00	0.00	0.00	0.24	0.01	0.00	0.00
74	0.00	0.00	2.74	0.06	0.00	0.00	0.30	0.01	0.03	0.01	0.00	0.00	0.00	0.00	0.00	0.00	0.21	0.02	0.00	0.00

E. coli **BW25113** ***Δicd ΔxylAB::*****Cm**^{R} **pCX-pL-*****xylAXB***$_{Cc}$ **pCL-*****xylCD***$_{Cc}$

Fig. 5e in text

**Time (h)**	**Ethanol g/L**		**Acetate g/L**		**Lactate g/L**		**Formate g/L**		**Succinate g/L**		**2-oxoglutarate g/L**		***D*-Xylose g/L**		**Pyruvate g/L**		***D*-Xylonate/ *D*-Xylonolactone g/L**		***D*-KDX g/L**	
	AVG	SD	AVG	SD	AVG	SD	AVG	SD	AVG	SD	AVG	SD	AVG	SD	AVG	SD	AVG	SD	AVG	SD
0	0.00	0.00	0.00	0.00	0.00	0.00	0.00	0.00	0.00	0.00	0.00	0.00	10.80	0.13	0.00	0.00	0.00	0.00	0.00	0.00
12	0.00	0.00	0.27	0.02	0.06	0.01	0.00	0.00	0.21	0.01	0.04	0.01	8.96	0.24	0.27	0.01	0.00	0.00	0.00	0.00
18	0.00	0.00	0.33	0.01	0.13	0.01	0.02	0.02	0.27	0.01	0.05	0.01	8.02	0.18	0.46	0.01	0.00	0.00	0.00	0.00
23	0.00	0.00	0.41	0.01	0.16	0.03	0.02	0.03	0.31	0.01	0.07	0.01	7.49	0.16	0.57	0.01	0.00	0.00	0.00	0.00
59	0.00	0.00	0.41	0.01	0.16	0.04	0.08	0.01	0.23	0.04	0.11	0.01	6.52	0.05	0.64	0.02	0.00	0.00	0.00	0.00

E. coli **BW25113** ***ΔxylAB::*****Cm**^{R} **pCX-pL-*****xylAXB***$_{Cc}$ **pCL-*****xylCD***$_{Cc}$

**Time (h)**	**Ethanol g/L**		**Acetate g/L**		**Lactate g/L**		**Formate g/L**		**Succinate g/L**		**2-oxoglutarate g/L**		***D*-Xylose g/L**		**Pyruvate g/L**		***D*-Xylonate/ *D*-Xylonolactone g/L**		***D*-KDX g/L**	
	AVG	SD	AVG	SD	AVG	SD	AVG	SD	AVG	SD	AVG	SD	AVG	SD	AVG	SD	AVG	SD	AVG	SD
0	0.00	0.00	0.00	0.00	0.00	0.00	0.00	0.00	0.00	0.00	0.00	0.00	10.95	0.07	0.00	0.00	0.00	0.00	0.00	0.00
21	0.00	0.00	0.25	0.01	0.08	0.02	0.00	0.00	0.24	0.03	0.00	0.00	9.53	0.03	0.25	0.01	0.00	0.00	0.00	0.00
25	0.00	0.00	0.35	0.01	0.13	0.01	0.00	0.00	0.29	0.01	0.00	0.00	8.97	0.24	0.33	0.01	0.00	0.00	0.00	0.00
29	0.00	0.00	0.38	0.04	0.17	0.01	0.00	0.00	0.33	0.01	0.00	0.00	8.62	0.25	0.40	0.01	0.00	0.00	0.00	0.00
37	0.00	0.00	0.40	0.01	0.23	0.01	0.03	0.00	0.34	0.02	0.00	0.00	8.12	0.13	0.47	0.01	0.00	0.00	0.00	0.00
53	0.00	0.00	0.44	0.01	0.25	0.01	0.05	0.00	0.26	0.02	0.00	0.00	7.70	0.02	0.42	0.01	0.00	0.00	0.00	0.00
59	0.00	0.00	0.44	0.01	0.23	0.02	0.06	0.03	0.24	0.02	0.00	0.00	6.86	0.06	0.40	0.02	0.00	0.00	0.00	0.00
